# Smarcb1 Loss Results in a Deregulation of esBAF Binding and Impacts the Expression of Neurodevelopmental Genes

**DOI:** 10.3390/cells11081354

**Published:** 2022-04-15

**Authors:** Amelie Alfert, Carolin Walter, Natalia Moreno, Viktoria Melcher, Monika Graf, Marc Hotfilder, Martin Dugas, Thomas Albert, Kornelius Kerl

**Affiliations:** 1 Department of Pediatric Hematology and Oncology, University Children’s Hospital Münster, 48149 Münster, Germany; amelie.alfert@ukmuenster.de (A.A.); natalia.morenogalarza@ukmuenster.de (N.M.); viktoria.melcher@ukmuenster.de (V.M.); monika.graf@ukmuenster.de (M.G.); marc.hotfilder@ukmuenster.de (M.H.); thomas.albert@ukmuenster.de (T.A.); 2 Institute of Medical Informatics, University of Münster, 48149 Münster, Germany; carolin.walter@ukmuenster.de (C.W.); martin.dugas@med.uni-heidelberg.de (M.D.); 3 Institute of Medical Informatics, Heidelberg University Hospital, 69120 Heidelberg, Germany

**Keywords:** Smarcb1, BAF complex, embryonic stem cells, chromatin remodelling, ChIP-seq

## Abstract

The murine esBAF complex plays a major role in the regulation of gene expression during stem cell development and differentiation. As one of its core subunits, Smarcb1 is indispensable for its function and its loss is connected to neurodevelopmental disorders and participates in the carcinogenesis of entities such as rhabdoid tumours. We explored how Smarcb1 regulates gene programs in murine embryonic stem cells (mESC) and in this way orchestrates differentiation. Our data underline the importance of Smarcb1 expression and function for the development of the nervous system along with basic cellular functions, such as cell adhesion and cell organisation. Using ChIP-seq, we were able to portray the consequences of Smarcb1 knockdown (kd) for the binding of esBAF and PRC2 as well as its influence on histone marks H3K27me3, H3K4me3 and H3K27ac. Their signals are changed in gene and enhancer regions of genes connected to nervous system development and offers a plausible explanation for changes in gene expression. Further, we describe a group of genes that are, despite increased BAF binding, suppressed after Smarcb1 kd by mechanisms independent of PRC2 function.

## 1. Introduction

Embryonic stem cells (ESCs) are able to self-renew as well as to differentiate into every cell lineage of the mature organism. Self-renewal, establishment and maintenance of pluripotency is governed by a core regulatory circuitry of factors such as Pou5f1, Nanog and Sox2 [1,2,3], in cooperation with additional genes such as *Klf4*, *Tbx3*, *Stat3* or *Esrrb* [4]. Further, ESCs are characterised by their unique epigenetic structure. They have a more open chromatin, and many genes that are essential for the regulation of ESC pluripotency and differentiation are marked by so-called bivalent domains, i.e., the simultaneous presence of the activating and repressive chromatin marks H3K4me3 and H3K27me3, respectively [5,6,7].

Gene expression is regulated by various mechanisms including DNA methylation, histone modifications and ATP-dependent chromatin remodelling by multi-protein complexes, such as the BAF complex [8,9]. In contrast to changes in DNA sequences, these epigenetic modifications are dynamic and reversible.

Histone acetylation, especially H3K27ac, is crucial for gene regulation and results in a more accessible chromatin structure. Therefore, it is found at active gene regions as well as enhancers [10,11,12]. In contrast, histone methylation can be activating and repressive. While H3K4me3 is associated with promoter regions of actively transcribed genes, H3K27me3 is a canonical repressive mark with a central role in dynamic gene regulation (e.g., in bivalent domains) [5,12,13,14,15,16].

The responsible histone methyltransferase (HMT) for the trimethylation of H3K27me3 is Ezh2, the catalytic subunit of the Polycomb Repressive Complex 2 (PRC2) [17]. H3K27me3 recruits the Polycomb Repressive Complex 1 (PRC1), a family of complexes which in turn cause the ubiquitination of H2A (H2AK119ub1), chromatin compaction and transcriptional repression [18,19,20,21]. PRC1/2 activity and targeting are modulated by the third class of epigenetic regulators, namely the ATP-dependent chromatin remodelling complexes. One of them, the SMARCA4 (BRG1)-associated BAF complex, is the mammalian counterpart of the yeast SWI/SNF complex [22]. As BAF complexes are combinatorically assembled by up to 15 distinct subunits encoded by 29 genes, specificity in different cell types and developmental stages is achieved [23,24,25]. There are three major groups of BAF complexes that are differentiated based on their subunit composition: (I) the canonical BAF complex (cBAF) and (II) the Polybromo-associated BAF complex (PBAF) containing SMARCA4 (aka BRG1) or SMARCA2 (aka BRM) as an ATPase subunit and share a core module of a SMARCC1/C2 heterodimer, SMARCE1, SMARCD1 and SMARCB1 [25,26,27]. In contrast, (III) the recently described non-canonical BAF complex (ncBAF) is characterised by specific subunits but notably lacks, amongst others, the core subunit SMARCB1 [28,29,30,31].

Additionally, a BAF-complex composition differs during developmental stages. The ESC-specific BAF complex (esBAF) is defined by a unique dependency on SMARCA4 as ATPase subunit, the incorporation of ARID1A, SMARCD1 or D2 and a SMARCC1/C1 homodimer [26,32,33]. This complex is tightly linked to the regulation of the core pluripotency network and to the LIF-activated STAT3 signalling pathway that maintains pluripotency [34]. Furthermore, esBAF has been shown to interact with PRC1 and PRC2. In general, the BAF and PRC are considered to be antagonistic, with the BAF complex causing direct and indirect eviction of PRC1/2 from the chromatin in an ATP-dependent manner [35,36,37]. However, they appear to function agonistically in ESC by co-repressing differentiation-associated gene loci, such as the *Hox* gene clusters [34,35].

During differentiation, the BAF-complex composition changes in a tightly regulated manner and a correct formation and function of BAF complexes is critical for the faultless development of the mature organism [38]. For example, a homozygous knockout of the core subunits *Smarca4* or *Smarcb1* in mouse models results in early embryonic lethality [39,40]. In addition, alterations in BAF complexes, e.g., by subunit mutations, can have severe consequences after embryogenesis. This notion is highlighted by the fact that an estimated 20% of all human cancers contain such mutations [23,41]. In rhabdoid tumours, a highly malignant paediatric cancer, more than 95% of patients harbour a homozygous loss of the core subunit SMARCB1 [42,43]. Further, BAF-complex subunit alterations (of cBAF and ncBAF) are linked to neurodevelopmental disorders, such as Coffin Siris Syndrome [44,45,46,47]. Nevertheless, the precise mechanisms on how Smarcb1 regulates gene programs in embryonic stem cells remain elusive. Therefore, we investigated the impact of an induced loss of Smarcb1 on the transcriptional and epigenetic landscape of murine ESC. We were able to show the irreplaceability of Smarcb1 for the regulation of genes associated with the nervous system development. Further, we investigated the impact of Smarcb1 on BAF complex function and unravelled the existence of a Smarcb1-dependent gene set that is silenced after Smarcb1 knockdown in a PRC2-independent manner.

## 2. Materials and Methods

### 2.1. Generation of the 81K1 ESC Line

**Scheme 1:**
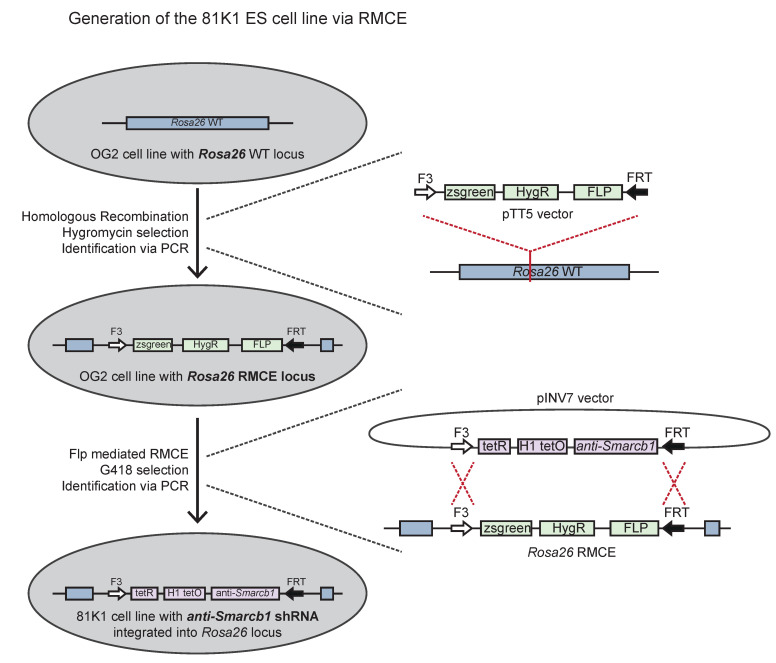
Generation of the 81K1 ESC line. Schematic overview of the generation of the 81K1 ESC line using RMCE. *Anti-Smarcb1* shRNA is inserted into the *Rosa26* locus and is expressed in a doxycycline-dependent manner.

We generated mESC with a specific and doxycycline-inducible knockdown of Smarcb1 and named these cells 81K1. For this purpose, a RMCE (recombinase-mediated cassette exchange) vector approach was used, which allowed a site-directed integration of a shRNA expression cassette in a previously modified genomic locus. In a first step, the acceptor vector pTT5 was integrated into the wild type *Rosa26* locus (*Rosa26* WT) of the parental OG2 cell line via homologous recombination. Clones with successful insertion were selected using hygromycin and further identified by PCR. Clone K1 was chosen for the following experiments based on PCR results as well as cell morphology. In a second step, the donor vector pINV7 was inserted into the modified rosa26 locus of clone K1 using Flp-mediated RMCE. pINV7 contained an anti-Smarcb1 shRNA (for sequences, see Supplementary Table S4) expressing cassette under the control of a doxycycline dependent promoter. Thus, anti-Smarcb1 shRNA is only expressed under the presence of doxycycline. Cells were selected using G418 and PCR analyses have been performed to ensure successful RMCE. For a schematic overview of the procedure, see Scheme 1.

### 2.2. Cell Culture

81K1 ESC were cultured at 37 °C and 5% CO_2_ on gelatine-coated plates in DMEM Glutamax-based medium (DMEM Glutamax (Invitrogen, Schwerte, Germany #31966021), 10% FBS superior (Biochrom, #S0615), 1% MEM non-essential amino acids (Sigma-Aldrich Chemie GmbH, Taufkirchen, Germany, #M7145-100ML), 1% beta-Mercaptoethanol (Gibco by Life Technologies, Schwerte, Germany, #31350-010), and 5 µg murine embryonic stem cell leukaemia-inhibitory factor (murine ESLIF) (PolyGene Transgenetics, Rümlang, Switzerland #PG-A1140-0100) per 500mL medium). All cell cultures were regularly tested on mycoplasma contamination. For knockdown experiments, cells were treated with 1 µg doxycycline (Sigma-Aldrich Chemie GmbH, Taufkirchen, Germany, #D9891-1G, dissolved in ddH_2_O) per ml medium every day during the running experiment.

### 2.3. RT-qPCR

For RT-qPCR, cells were harvested using Trypsin 0.05% EDTA (Gibco by life technologies, #25300054), washed with DPBS, pelleted and snap-frozen on dry ice. RNA was isolated by dissolving pellets in 1 mL TRIzol^®^ (LifeTechnologies, #15596018) and adding 200 µL Chloroform (Sigma-Aldrich Chemie GmbH, #288306). After incubation and centrifugation, the RNA-containing phase was collected and mixed with 500 µL isopropanol (SAV Liquid Production GmbH, Flintsbach am Inn, Germany, #ISOP-5000-100-1). After incubation and centrifugation, the remaining RNA pellet was washed in 1 mL 75% absolute pure ethanol (Applichem, Darmstadt, Germany, #A4230-1000PE) and dried before being resuspended in 50 µL DEPC water (Invitrogen, #AM9915G). Quantity and quality of RNA samples were assessed using the NanoDrop 2000 (ThermoFisher, Waltham, MA, USA, ND-2000) to ensure sufficient purity for RT-qPCR experiments.

1000 ng RNA was transcribed into cDNA using the PrimeScript™ RT Reagent Kit with gDNA Eraser (TaKaRa, Saint-Germain-en-Laye, France, #RR047B) according to the manufacturer’s instructions.

RT-qPCR was performed using 5 µL Power SYBR Green PCR Master Mix (Life Technologies, #4367659), 2 µL PrimerMix (primer concentration 100 µM), 1 µL cDNA and 2 µL ddH_2_O per well of the 96-well reaction plate (Applied Biosystems, Waltham, MA, USA, #4346907). RT-qPCR was performed using the BioRad C100 Thermal Cycler (BioRad Laboratories, Munich, Germany) according to our laboratories standard protocol (50 °C for 2 min, 95 °C for 10 min, 40 cycles with (I) 95 °C for 15 s, (II) 60 °C for 1 min and (III) detection). Quantification of RT-qPCR results was performed using the ΔΔCt method, implemented in the BioRad CFX manager 2.1; ΔCt values were calculated by using *Rpl3* as the endogenous control, ΔΔCt values by comparison were with an untreated (Smarcb1 positive) sample. Statistical analyses were performed with GraphPad Prism 7 for Windows. For oligonucleotides’ sequences, see Supplementary Table S3.

### 2.4. Western Blot

Pellets for the Western blot were resuspended and incubated in RIPA buffer (50 mM HEPES KOH (Sigma-Aldrich Chemie GmbH, # H3375), pH 7.4; 1 mM EDTA (Carl Roth GmbH&CoKG, Karlsruhe, Germany, #8043.2), pH 8.0; 0.5 M LiCl (Sigma-Aldrich Chemie GmbH, # 62476); 1% IGEPAL CA-630 (Sigma-Aldrich Chemie GmbH, # 18896); 0.7% NaDOC (Sigma-Aldrich Chemie GmbH, # D6750), centrifuged and the supernatant was collected. In a next step, protein concentrations were measured colorimetrically by a Bradford assay with Coomassie Brilliant Blue G-250 (Bio-Rad Life Science, Munich, Germany, #1610406) and a BSA-based standard curve (Fisher Scientific, Waltham, MA, USA, #15,260,037 diluted in ddH_2_O). O.D. values were measured at 595 nm in the Multiscan Ascent (Thermo Electron Corporation, now Thermo Fisher scientific, Waltham, MA, USA).

SDS-Polyacrylamide gel electrophoresis was run in 10–15% acrylamide gels (10% for Smarca4, Ezh2, Smarcb1, b-Actin and 15% for histones) with 20 µg protein per sample. Blotting was performed using the Trans-Blot^®^ Turbo System (BioRad, #1704150) and methanol-activated PVDF membranes (methanol: Carl Roth GmbH&CoKG, # 8388.5; PVDF membranes: BioRad, #1620177). Membranes were blocked with 5% milk (powdered milk (Carl Roth GmbH&CoKG, # T145.2) dissolved in TBS-Tween) and incubated with primary antibodies overnight (Smarcb1: BD transduction laboratories, #612110, 1:1000; Ezh2: Cell signalling, #5246S, 1:4000; Smarca4: abcam, #ab110641; H3K27me3: Merck KGaA, #07-449, 1:5000; H3K27ac: Abcam, #ab4729, 1:5000; H3K4me3: Diagenode, #pAb-003-050, 1:5000). Incubation with secondary antibodies (Peroxidase-conjugated anti-mouse, Jackson Immuno Research, Hamburg, Germany, #115-035-044; Peroxidase-conjugated anti-rabbit, Jackson Immuno Research, #111-035-045, both 1:5000) was performed for 1 h at room temperature. For visualisation, PerkinElmer’s Western Blot ECL Pro Solution (PerkinElmer, Rodgau, Germany, #NEL120001EA) was added and signals were detected by the Fusion Western Blot Detector (Vilber). Western blot bands were semi-quantitatively analysed using ImageJ 1.51f. All antibody signals were normalised to b-Actin. Raw data were processed using Microsoft Excel 2010 (Microsoft Corporation, Redmond, WA, USA) and statistical analyses were performed with GraphPad Prism 7 (STATCON GmbH, Witzenhausen, Germany).

### 2.5. Co-IP Experiments

For co-immunoprecipitation (co-IP) experiments, 81K1 ESC cells were grown to near-confluence on 20 cm culture dishes, as described above. For knockdown experiments, cells were treated with 1 µg doxycycline per ml medium per day for three days. Afterwards, cells were washed with cold PBS and lysed on ice with 1 mL of cold GENNT buffer (5% glycerol, 5 mM EDTA, 0.2% NP-40, 150 mM NaCl, 50 mM Tris–HCl pH 8.0, 0.5 mM PMSF, freshly added 2 µg/mL pepstatin A, leupeptin and aprotinin, respectively, 10 mM sodium fluoride and 2 mM sodium orthovanadate). Lysate was scraped off plates, sheered using a 27 gauge needle several times and centrifuged at 10,000× *g* for 15 min at 4 °C. Protein concentration was measured using the colorimetric Bradford method.

For immunoprecipitation, 50 µL A/G PLUS agarose beads (Santa Cruz Biotechnology, Heidelberg, Germany, #sc-2003) were coupled for at least 2 h at 4 °C with anti-Brg1 (H-88) (Santa Cruz Biotechnology, #sc-10768) or anti-Baf155 (R18) (Santa Cruz Biotechnology #sc-9746) in GENNT buffer. Subsequently, cell lysate was added (1 mg) and incubated at 4 °C on a rocker platform overnight. Then, the beads were washed three times with GENNT buffer and boiled with 2X SDS loading buffer. Of the eluted material, 25 µL were loaded onto 10% gels and analysed by SDS–PAGE. Input represents a 10% of the cell lysate. SDS-PAGE and Western blot were performed as described above with the following antibodies: Primary antibodies: Brg1(N-15) (Santa Cruz Biotechnology, #sc-8749, 1:1000), Baf155 (D7F8S) (Cell Signaling Technology, Koblenz, Germany #9053, 1:1000), Smarcb1 (Y-7) (Santa Cruz Biotechnology, #sc-101161, 1:1000), α-Tubulin (B-7): (Santa Cruz Biotechnology, #sc-23948, 1:1000). Secondary antibodies: Peroxidase-conjugated anti-mouse (Jackson Immuno Research, #115-035-044, 1:2000) and Peroxidase-conjugated anti-rabbit (Jackson Immuno Research, #111-035-045, 1:2000).

### 2.6. Evaluation of Cell Growth and Cell Viability: MTT Assay and Doubling Time

Doubling time of 81K1 ESC was assessed in three separate time windows: 0 to 3 days, 4 to 6 days and 7 to 9 days, respectively, to allow optimal growth conditions. Cells were seeded in equal density for Ctrl and Dox samples and counted every day. Doubling time was calculated using the following formula: $Doubling\;time=\frac{t2\;-\;t1}{3.32\;\times \;\left(logn2\;-\;logn1\right)}$ with *t* = duration of treatment in h and *n* = number of counted cells.

For the MTT assay, cells were seeded on 96-well plates 24 h prior to first treatment. Medium was refreshed every two days for both conditions. For measurement, 10 µL MTT solution (Merck KGaA, Darmstadt, Germany, #CT01) was added to the medium and mixed carefully by tossing the plate. After 3.5 h incubation at 37 °C and 5% CO_2_ to allow formazan crystal forming, 100 µL Isopropanol (SAV Liquid Production GmbH, #ISOP-5000-100-1) containing 0.04 N HCl (Honeywell Fluka™, Bucharest, Romania, #71763) was used to dissolve crystals. Plates were measured at 550 nm and 630 nm (reference wavelength) using the Multiscan Ascent (Thermo Electron Corporation). To confirm our data, manual counting experiments have been performed with an additional clone, the 81K4 ESC line. Cells were plated in the same density, first treated at day 0 and counted every 24 h. Raw data were processed using Microsoft Excel 2010 (Microsoft Corporation) and statistical analyses were performed with GraphPad Prism 7 (STATCON GmbH).

### 2.7. RNA-Seq

For RNA-seq, cells were cultured and treated as described above. For RNA isolation, the Rneasy Mini Kit (Qiagen, Hilden, Germany, #74104) was used according to the manufacturer’s instructions. Quality and quantity of isolated RNA were evaluated using the NanoDrop 2000. Libraries were prepared and sequenced (~20 M single reads per sample) using the Illumina Next-Seq 500 sequencing platform (high-output Kit, 75 Cycles v2 Chemie) at the Genomics Core Facility (University Hospital Münster, Münster).

The raw fastq files were aligned against the murine reference genome mm10 with the alignment algorithm STAR v2.7.0c [48]. We used the R/Bioconductor packages GenomicRanges [49] and Rsamtools [50] to create count tables per gene and the sample, and calculated differentially expressed genes between Smarcb1 knockdown samples and its matching control data with the R/Bioconductor package DESEQ2 [51]; for the 72 h knockdown set, the 48 h control data were substituted. The resulting gene lists were filtered with R [52] to only include genes with an adjusted *p*-value <0.05 and an absolute log fold change >0.58, and subsequently exported for use with QIAGEN’s Ingenuity Pathway Analysis (IPA) (QIAGEN Inc. (Hilden, Germany), https://www.qiagenbioinformatics.com/products/ingenuity-pathway-analysis, accessed on 31 December 2021, [53,54]) ).

Cells were evaluated at three timepoints: 48 h, 72 h and 240 h after *Smarcb1* knockdown. Dataset overlaps have been calculated using Ingenuity Pathway Analysis (IPA, Qiagen) and Venn diagrams (Supplementary Figure S2A) were drawn manually in Illustrator (Illustrator 2021, Adobe Inc., San Jose, CA, USA).

### 2.8. ChIP-Seq for Ezh2, H3K27ac and H3K4me3

ChIP-seq experiments for Ezh2, H3K27ac and H3K4me3 were performed 72 h after kd induction. Cells were fixed in 1% formaldehyde solution (Sigma-Aldrich Chemie GmbH, #F8775) for 9 min and quenched with 0.125 M Glycine (Carl Roth GmbH & CoKG, # 3790.2) for 5 min at room temperature. Chromatin extraction was performed using lysis buffers 1–3 (see Supplementary Table S2). Samples were thoroughly mixed with the lysis buffer, incubated on ice (15 min with lysis buffer 1, 10 min with lysis buffer 2), centrifuged and the supernatant was discarded. For chromatin fragmentation, a two-step sonication protocol was utilised. Samples were first sonified at 40% amplitude for 9 cycles (30 s ON, 30 s OFF) using a Branson Sonifier^TM^ (Fisher Scientific GmbH) and secondly at a high output for 15 min (30 s ON, 30 s OFF) in a Bioruptor^®^ (Diagenode, Seraing, Belgium). Before freezing, samples were mixed with glycerol (AppliChem GmbH, A1123,2500) to an end concentration of 5.5%. Fragmentation sufficiency was confirmed with an Ethidium bromide gel electrophoresis.

For chromatin immunoprecipitation, IgG Dynabeads^TM^ (Novex by Life Technologies, #10004D) were linked to the required antibody (H3K27ac: abcam, #ab4729; H3K4me3: Diagenode, #pAB-003-050; Ezh2: Cell Signalling, #5246S; IgG: Novusbio, #NBP2-24891) after blocking with BSA (New England BioLabs, Ipswich, MA, USA, #B9000S). Per reaction, 15 µL antibody-linked beads, 100 µL Chromatin Extract and 900 µL ChIP assembly buffer (10 mM Tris-HCl pH 8.0, 140 mM NaCl (Carl Roth GmbH & CoKG, #3957.2), 1 mM EDTA pH 8.0, 0.5 mM EGTA (Sigma-Aldrich Chemie GmbH, #E4378) pH 8.0, 1% Triton (AppliChem GmbH, A4975,0500), 0.135% NaDOC, and 1 X cOmplete^TM^ (Sigma-Aldrich Chemie GmbH, # 11697498001) were incubated overnight. After washing, ChIP samples were incubated in ChIP elution buffer (50 mM Tris-HCl pH 8.0, 10 mM EDTA, pH 8.0, 1% SDS (Sigma-Aldrich Chemie GmbH, #74255), and Rnase A (Invitrogen, #AM2272) as well as Proteinase K (Sigma-Aldrich Chemie GmbH, #3115828001) were added stepwise. All samples were purified using the QIAquick PCR Purification Kit (Qiagen, #28104) according to the manufacturer’s instructions.

For evaluation of ChIP efficiency prior to sequencing as well as ChIP validation, RT-qPCR was performed with the settings described under RT-qPCR. By using Ct values of diluted inputs (2%, 0.4 %, 0.08%) to create a standard curve, antibody- and primer-specific input recoveries were calculated using the Excel function, “variation”.

Libraries were prepared and sequenced (~20 M single read per sample) using the Illumina Next-Seq 500 sequencing platform (high-output Kit, 75 Cycles v2 Chemie) at the Genomics Core Facility (University Hospital Münster, Münster).

### 2.9. ChIP-Seq for Smarca4 and H3K27me3

ChIP-seq experiments for Smarca4 and H3K27me3 have been performed by Active Motif. Cultured cells (72 h after kd induction) were fixed in our laboratory according to Active Motif’s instructions before being snap-frozen and shipped on dry-ice. Chromatin immunoprecipitation (Smarca4: Abcam, ab110641, H3K27me3: Active Motif, #39155), library preparation and sequencing were performed by Active Motif. Resulting raw data were processed in parallel to the datasets obtained from the Core Facility Genomics for Ezh2, H3K4me3 and H3K27ac. For validation, independent crosslinked cells were sent to Active Motif, where ChIP-qPCR was performed.

### 2.10. Analysis of ChIP-Seq Data—Alignment and Peak Calling

Briefly, the resulting sequencing data was processed using the Galaxy platform [55], including both quality and adapter trimming, and a standard alignment against the murine reference genome mm10.

The resulting alignment was filtered with samtools 1.9 [56] to include only uniquely mapped reads with less than two mismatches. Subsequently, peak calling was conducted with the algorithm MACS2 [57] for the transcription factors Smarca4 and Ezh2, and the program SICER [58] for the histone modifications H3K27me3, H3K27ac and H3K4me3. Default parameters were kept for the MACS2 algorithm’s peak calling; for SICER, a window size of 200 bp, a gap size of 600 bp and default parameters otherwise were used. The SICER-based peak lists were filtered further based on their *p*-value, with a cut-off of 10^−5^ for H3K27ac and 10^−10^ for the remaining histones. For both MACS2 and SICER, two different peak calling strategies were employed to identify peaks for a single chosen condition, and differential peaks between two conditions. The basic SICER routine was used for the regular peak to call in the histone modification samples, while SICER-df was chosen to identify differential peaks. In all SICER analyses, the input controls were supplied for all respective conditions as well to serve as a background or control sample. This general approach was mirrored with MACS2 for the analysis of Smarca4 and Ezh2. For the first strategy, a standard peak calling was performed for each chosen antibody. During this regular peak calling, input samples were supplied as control for the MACS2 callpeak function, while differential peaks between two conditions were identified through a second, separate analysis by using the second condition in place of the control input for the MACS2 algorithm and vice versa. We calculated the overlap of both peak sets for each antibody, which were called differential peaks or “DECREASE” and “INCREASE” in the following sections, with the BEDTools program suite [58]. Thus, any peak region classified as “INCREASE” or “DECREASE” showed both a significant enrichment against their respective input control (i.e., a regular peak for the condition), and a significant enrichment between both conditions. We also identified common peaks between two antibody ChIP-seq tracks by calculating the overlap between the corresponding standard peak sets for both SICER and MACS2. Thus, all further downstream analyses were performed on standard peaks per condition (“control”/”ctrl” or “knockdown”/”kd”), common peaks between conditions (“COMMON”/”COM”), differential peaks (“DECREASE”/”DECR” or “INCREASE”/”INCR”), or a combination of these three sets.

### 2.11. ChIP-Seq Visualisation in Peak Profiles

Annotations and localisations of peaks were added with the R/Bioconductor packages biomaRt [59] and ChIPseeker [60], and HOMER’s annotation routine [61]. Additional visualisations and quality controls in form of antibody binding profiles were conducted with basic plot functions in R [52]. For each binding profile, the raw ChIP-seq data’s coverage was computed with the R/Bioconductor package GenomicAlignments [49] for intervals of ±2.5 kbp length, centred on each individual peak’s centre. The corresponding average coverage profile per antibody peak set was computed with R and smoothed with the LOESS algorithm [62].

### 2.12. Gene Ontology (GO) Analysis

For GO analysis of RNA-seq data (*Smarcb1* knockdown, *Smarca4* knockdown), genes were separated by their expression and up- and downregulated genes were analysed separately. For GO-term identification, ToppFun (part of the ToppGene Suite [63] was used. For gene names not found by the database, alternatives for missing symbols have been searched and used if applicable. Regarding statistical calculations and standard settings were kept (*p* value method: probability density function, correction for multiple testing: FDR, *p* value cut-off 0.05). The resulting lists for biological functions were transferred into Excel and per term, categories were assigned manually (*Adhesion, Cell Organisation, Cell Cycle, Chromosome Organisation, Protein/RNA/DNA, Metabolism, Response to Stimulus, Nervous System, Synapse, Development* (*without Neuron*)). For terms with more than one suitable category, both were used, and terms without a suitable category were summarised as *Others*.

The analysis of genes with a change in antibody binding in ChIP-seq was performed analogously. Gene sets were analysed individually, separated (1) by antibody and (2) by increase/decrease. For further procedure, see above.

**Figure 1:**
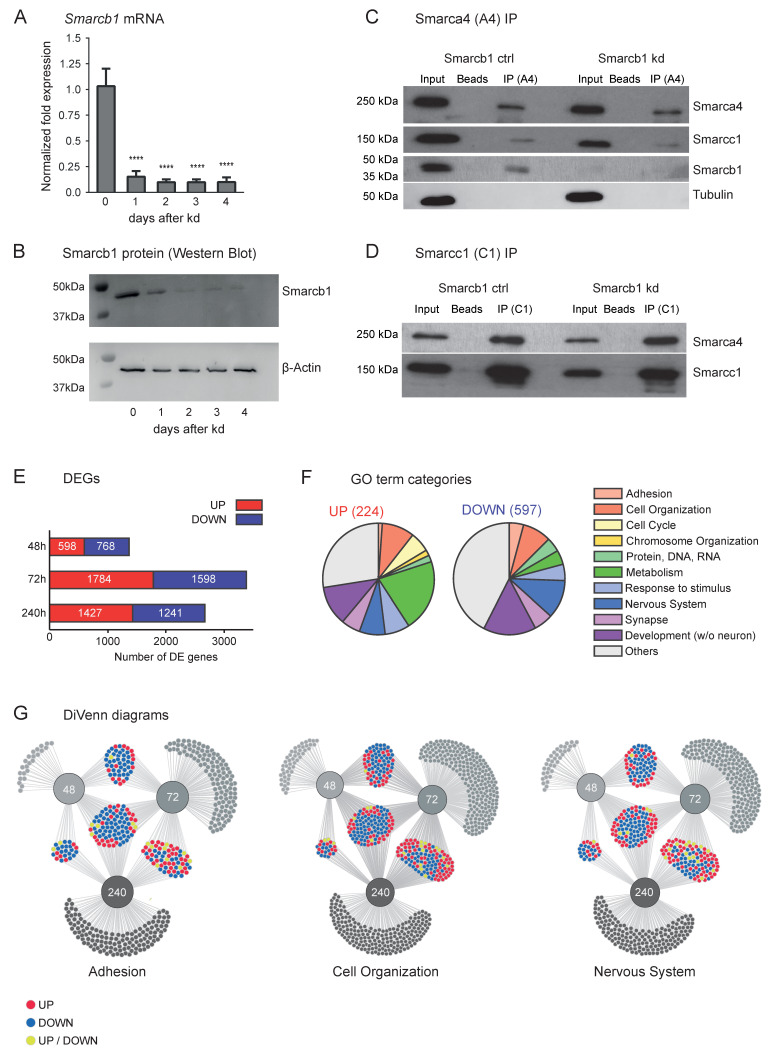
*Smarcb1* kd impacts expression of *cell adhesion*-, *cell organisation*-, *neuron system*- and *development*-associated genes (**A**) *Smarcb1* expression on mRNA level measured by RT-qPCR in n = 3 biological replicates. Error bars represent SEM, **** = *p* < 0.0001. (**B**) Smarcb1 signal in a representative Western blot with b-Actin as loading control. (**C**,**D**) Immunoprecipitation (IP) of BAF-complex core units Smarca4 (A4) and Smarcc1 (C1) from nuclear extracts of Smarcb1-positive (Smarcb1 ctrl) and Smarcb1-kd ESC. Tubulin was used as a loading control. (**E**) Number of differentially expressed genes (DEG) (*p* < 0.05 and abs. log2FC > 0.58) after *Smarcb1* knockdown at indicated time points in RNA-seq. (**F**) GO terms assigned to up- and downregulated genes 72 h after *Smarcb1* knockdown. GO terms were manually separated into categories and overall number (in parenthesis) as well as distribution are depicted here. (**G**) Venn diagrams depict the overlap of DEG at three time-points after *Smarcb1* kd (48 h, 72 h, 240 h) belonging to categories *Adhesion, Cell Organisation* or *Nervous system.* Gene lists were created by using representative GO-term gene lists (see Methods). Genes uniquely deregulated at one time point are greyed-out for simplification.

For Figure 1G, representative GO terms have been chosen to create a gene list for categories *Adhesion* (GO:007417—“cell adhesion”), *Cell organisation* (GO:0007010—“cytoskeleton organization”, GO:0030030—“cell projection organization”, GO:0000902—“cell morphogenesis”) and *Nervous System* (GO:0022008—“neurogenesis”, GO:0007417—“central nervous system development”). Gene lists were obtained from the Mouse Genome Informatics website (http://www.informatics.jax.org, accessed 3 June 2020). Duplicates were deleted, resulting gene lists were merged with expression data and filtered for significant genes (*p* < 0.05, abs. log2FC > 0.58) individually for datasets of 48 h, 72 h and 240 h *Smarcb1* knockdown. Overlaps of these gene lists were analysed and visualised using DiVenn [64].

### 2.13. Comparison of Smarca4 and Smarcb1 Knockdown Data

Smarca4 kd data were obtained from Ho et al. [34] and downloaded from the GEO website using GEO2R with recommended standard settings. Gene lists were uploaded to IPA (QIAGEN Inc.) and merged and compared with the Smarcb1-kd dataset also uploaded to the platform.

For presentation of resulting data in a heat map, they were summarised in one Excel sheet (column 1: gene name, including all genes with *p* < 0.05 in one or both datasets, column 2: log2FC after *Smarcb1* knockdown (in case of *p* > 0.05 or abs. log2FC < 0.58 replaced by “0”), column 3: log2FC after *Brg1* knockdown (in case of *p* > 0.05 or abs. log2FC < 0.58 replaced by “0”)). Heatmaps were created with the help of heatmap2 on the Galaxy platform [55]. The following settings were used: label columns, not rows; colour groups blue to white to red; do not scale data, no clustering, all others: standard setting. The resulting heatmap was further processed in illustrator (Illustrator 2021, Adobe Inc.) to apply the chosen colour code.

After matching datasets of *Smarca4* and *Smarcb1* knockdown, genes were subdivided into six categories: core esBAF-repressed genes/core esBAF-activated genes/Smarcb1-repressed, not Smarca4-repressed genes/Smarcb1-activated not Smarca4-activated genes/Smarca4-repressed, not Smarcb1-repressed genes/Smarca4-activated, and not Smarcb1-activated genes. All included genes were required to have a *p* value < 0.05. For thresholds for log2FC (if used), see Supplementary Table S1.

### 2.14. Combining RNA-Seq and ChIP-Seq Data

The annotated ChIP-seq peak sets were filtered with R [52] and the BedTools program suite [65] to exclude all peaks that did not have any overlaps with any gene or TSS region (+/− 1 kbp) based on GENCODE’s GRCm38 reference, version M23 (Ensembl 98) [66]. The remaining ChIP-seq peaks were assigned to the overlapping gene/TSS intervals in R and merged with the RNA-seq data based on their gene IDs.

Resulting data were summarised in individual Excel sheets and heatmaps were created with the help of Galaxy Europe, as described above. Resulting heatmaps (labelling, colours) were processed in Illustrator (Illustrator 2021, Adobe Inc.).

### 2.15. Permutation Tests and Antibody Enrichment

We used permutation tests to compare antibody binding patterns in Smarcb1-activated genes. To this end, we extracted all genes that were significantly downregulated 72 h after Smarcb1 kd with an adjusted *p*-value < 0.05 and a log fold change < −0.58, and showed a parallel differential ChIP-seq peak signal for Smarca4. This basic gene set was split into groups depending on the Smarca4 peak category (DECREASE or INCREASE); for both of the resulting subgroups, the number of genes with a concurrent ChIP-seq signal (control, knockdown, DECREASE, INCREASE or COMMON) for any of the chosen antibodies (Ezh2, H3K27me3, H3K27ac, and H3K4me3) was counted with basic R functions. For each antibody and peak set combination, a null distribution was created by randomly distributing the total number of respective genes with antibody-related ChIP-seq signal for 1.000.000 times, splitting the results into random DECREASE and random INCREASE sets relative to the basic gene set’s DECREASE/INCREASE ratio, and counting the respective gene numbers in both sets. An antibody peak–set combination was called significantly enriched in one partition of the basic gene set if the actual number of genes with antibody ChIP-seq signal was in the 5 % quantile of the random distribution. A two-sided test was used, i.e., the lowest or highest 2.5 % of random shuffles were considered and corrected for multiple testing using the Bonferroni method [67]. Any antibody peak–set combination with a higher or lower gene count per basic gene set partition than randomly occurring in 1.000.000 permutations was called “highly” significant.

A similar permutation test approach was used to determine enrichments of binding patterns of Smarcb1-activated genes in Smarca4-activated or -repressed subgroups. All calculations and permutations were conducted with basic R functions.

Resulting dot plots were further processed in Illustrator (Illustrator 2021, Adobe Inc.) to adjust colour schemes. In figures, aquamarine circles represent a positive correlation (genes are more likely to be bound by the antibody of interest if they are in the tested group), while red circles represent a negative correlation (genes are less likely to be bound by the antibody of interest if they are in one particular group). The size of circles symbolises significance level: (I) small, grey circles: no significance, (II) small, coloured circles: significant, (III) large, coloured circles: highly significant.

### 2.16. Enhancer Analysis

For the enhancer analysis, predicted cis-regulatory elements for murine ESC cells were downloaded from the Mouse Encode Project [68]. We used the UCSC Genome Browser’s LiftOver tool [69] to convert the mm9-based specific murine ESC intervals to mm10 and extended each enhancer interval to a length of 3001 bp, centred on the original 1 bp location. The BEDTools program suite [65] and R were used to calculate the overlaps between the murine ESC enhancers and the ChIP-seq peak lists, which were subsequently annotated with ChIPseeker [60] and merged with the RNA-seq data based on their nearest gene (up to a maximum distance of 100.000 bp).

Violine plots were created with the help of the Galaxy platform [55] using ggplot2 [70] with standard settings (as suggested by Galaxy). Resulting violine plots were processed in Illustrator (Illustrator 2021, Adobe Inc.) to adjust colours and labelling.

## 3. Results

### 3.1. Smarcb1 Regulates Gene Networks Involved in the Development of the Nervous System, Cell Organisation and Adhesion

We have established a conditional knockdown (kd) system for Smarcb1 in the mESC line OG2. Upon administration of doxycycline (Dox), these cells, termed 81K1, express *Smarcb1* shRNA, which result in a time-dependent and gradual decrease in *Smarcb1* mRNA as well as protein (Figure 1A,B, Supplementary Figure S1A). Even if Smarcb1, a core subunit of the BAF complex, is lost, the integrity of the BAF complex remains intact. This was confirmed by immunoprecipitation (IP) experiments in Smarcb1-positive and Smarcb1-kd cells. Core subunits Smarca4 and Smarcc1 remain tightly associated. As expected, Smarcb1 cannot longer be detected in the BAF complex after kd induction (Figure 1C,D).

Since Smarcb1 is known to directly interact with the PRC2 subunit (and H3K27me3 writer) Ezh2 as well as with several histone marks, we examined the effects of Smarcb1 kd on the levels of Ezh2 (mRNA and protein), histone marks H3K27me3, H3K27ac and H3K4me3 (Supplementary Figure S1B,D). However, neither Ezh2 nor histone marks were significantly affected in their global levels. Nevertheless, a clear effect of Smarcb1 kd on the proliferation and cell growth of 81K1 cells was observed: it resulted in a reduced growth of kd cells while Dox-treatment of the parental OG2 cell line had no effect. This was confirmed in an additional clone, the 81K4 cell line (Supplementary Figure S1E,G).

Next, we explored changes in gene expression caused by the loss of Smarcb1. Deep sequencing of RNA (RNA-seq) was performed on RNA isolated from untreated control cells as well as Smarcb1-kd cells (48 h, 72 h or 240 h after induction of the knockdown). At all timepoints, a substantial number of genes are either upregulated or downregulated, underlining the concept of esBAF operating both in gene activation and repression (Figure 1E). In addition, there is a considerable overlap between deregulated genes at all timepoints with 239 genes being upregulated and 337 genes being downregulated at all three examined timepoints (Supplementary Figure S2A).

Furthermore, gene ontology (GO) analyses were performed for all deregulated genes (*p* < 0.05, abs. log2FC > 0.58) 72 h after kd induction. The resulting terms were further classified into categories (emphasised by italicising) (Figure 1F and Supplementary Figure S2C). Both gene sets (up- and downregulated genes) share the differential expression of genes associated with *Nervous System*. Among the top 20 of the most significant terms are “neurogenesis” and “neuron differentiation”. However, the deregulation of *Nervous System*-related genes is even more prominent in the downregulated genes dataset, suggesting the importance of the esBAF complex to activate those genes. The same is true for GO terms connected to *Development.*

Even if the groups share many terms, they differ considerably in others: downregulated genes can partly be summarised by terms connected to *Adhesion*. These terms (e.g., “biological adhesion” and “cell adhesion”) are among the most frequent as well as the most significant terms of the GO analysis. In contrast, terms connected to upregulated genes are summed up by categories such as *Metabolism* (e.g., “lipid catabolic process”), while *Adhesion*-related terms are of minor importance. Further, they are marked by terms that are part of categories such as *Cell Cycle* and *Chromosome Organisation* and which barely occur when examining downregulated genes (Figure 1F and Supplementary Figure S2C). Importantly, genes connected to *Adhesion*, *Cell Organisation* and *Nervous System* are changed at all timepoints of our analysis and show a notable overlap. Most of these overlapping genes are stably up- or downregulated, but some are deregulated in different directions at different timepoints, indicating a time-dependency of changes after Smarcb1 kd (Figure 1G).

Although terms connected to differentiation are amongst the most significantly enriched GO terms, key pluripotency genes such as *Sox2, Pou5f1* and *Nanog* remain unchanged in their expression within the first days after Smarcb1 kd. However, other key factors such as *Tbx3* and *Klf4* are significantly downregulated at all timepoints (Supplementary Figure S2B).

### 3.2. The Regulation of Nervous System-Related Genes Is More Dependent on Smarcb1 Than on Smarca4 Function

In shRNA-based experiments, Ho et al. elucidated the effects of a Smarca4 knockdown on mESC [34]. Although it is known that BAF-complex subunits serve different functions, the unique implications of their loss for the complex’s function are still poorly understood. Therefore, we aimed to unravel the differences and similarities in gene regulation of the two core BAF complex subunits Smarca4 and Smarcb1. GO analysis of the Smarca4 kd dataset [34] resulted in 284 terms connected to upregulated genes and 689 terms related to downregulated genes (Supplementary Figure S2D). Both include terms of the categories *Development* as well as *Nervous System,* similar to results after Smarcb1 kd. Additionally, the upregulated genes can be connected to *Cell Cycle* as well as *Chromosome Organisation*. In contrast, the downregulated terms are dominated by the topics *Metabolism* and *Cell Organisation*. All of these categories contain the most frequent and the most significant terms (Supplementary Figure S2D).

**Figure 2:**
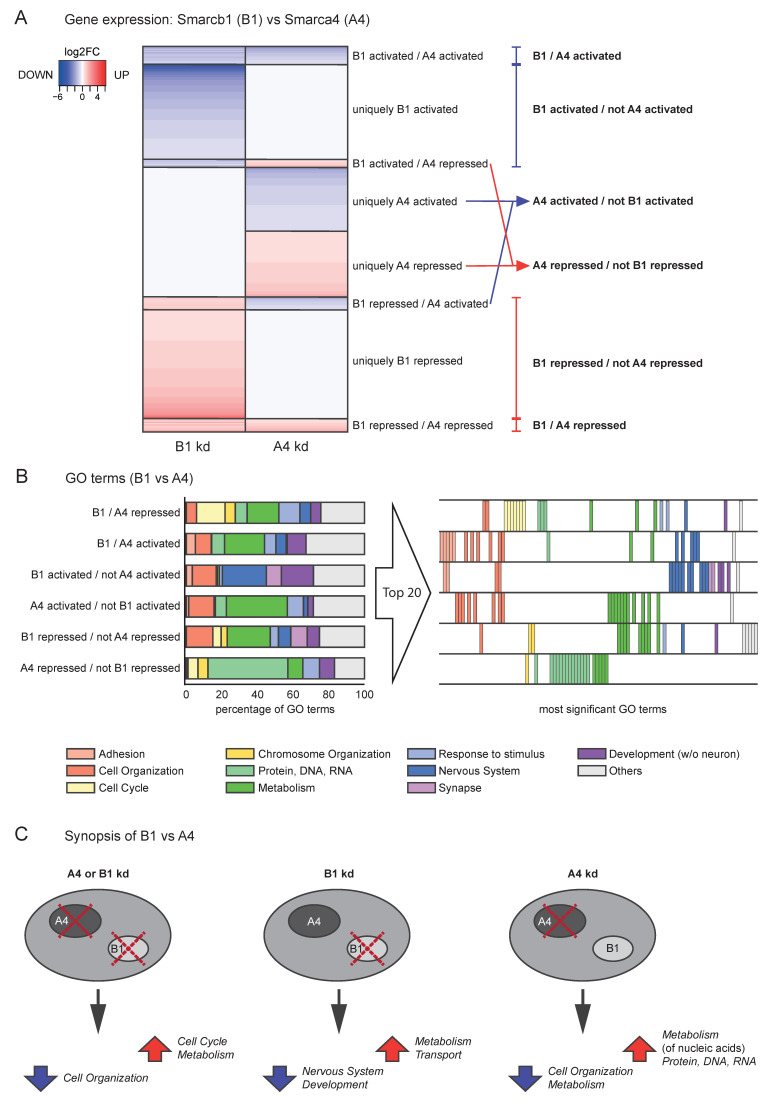
Comparison of gene expression after *Smarcb1* and *Smarca4* knockdown reveals differences between esBAF core components (**A**) Heatmap comparing gene expression data from our kd dataset (*Smarcb1* kd, 72 h) versus data obtained from Ho et al. (Ho et al., 2011) (*Smarca4* kd, 72 h). Only genes with *p* < 0.05 and an abs. log2FC > 0.58 in at least one dataset were included. Genes with *p* > 0.05 and/or an abs. log2FC < 0.58 are displayed in white, upregulated genes in red, downregulated genes in blue. Genes were further subclassified by their expression, as indicated in the figure. Group definition was set spaciously to include genes that show limited expression changes after short term kd (72 h) but are of relevance for cells’ function. Thresholds for log2FC were set according to Supplementary Table S1 (**B**) GO terms assigned to genes belonging to groups defined in (**A**). Left-hand part summarises GO-term classification into categories, right-hand part only includes GO terms within the top 20 (ordered by *p* value) in at least one dataset. (**C**) Synopsis of our findings concerning gene expression changes after *Smarca4* or *Smarcb1* kd.

To understand the different implications of Smarca4 (A4) versus Smarcb1 (B1) knockdown, we compared our RNA-seq dataset (Smarcb1 KD) with the microarray dataset (Smarca4 KD) (both 72 h after kd induction). Out of over 5000 genes being deregulated in at least one dataset, only a minority are changed in the same direction (Figure 2A). For further studies, we used the overlaps summarised in Figure 2A (all genes with *p* < 0.05, thresholds for log2FC are given in Supplementary Table S1) and performed a GO-term analysis (Figure 2B).

The B1/A4-repressed genes dataset is dominated by genes associated with *Cell Cycle* (e.g., “regulation of cell cycle”). Additionally, *Metabolism*-associated genes occur frequently but with less predominance in the top 20 of most significant terms. The B1-/A4-activated genes are highly connected to *Adhesion* (e.g., “cell adhesion”, “cell biological adhesion”) and *Cell Organisation* (e.g., “cell morphogenesis”). In addition, striking differences can be observed between the other groups: *Nervous System* (e.g., “neurogenesis”) and *Development* (e.g., “positive/negative regulation of developmental process”) are most prevalent when analysing B1-activated, not A4-activated genes. In contrast, A4-activated, not B1-activated, genes are scarcely related to those topics and cluster into categories such as *Cell Organisation* (e.g., “actin filament organization”) and *Metabolism* (including “lipid metabolic process”). B1-repressed, not A4-repressed, genes are in part connected to *Metabolism* (again including “lipid metabolic process”) but also to transport-related terms such as “ion transport”. A4-repressed, not B1-repressed, genes are dominated by terms associated with the metabolism of nucleic acids (e.g., “DNA metabolic process”) and other nucleic acid-related terms, such as “regulation of RNA splicing” (Figure 2B, summary in Figure 2C).

In summary, our data support the concept of subunit-specific effects of esBAF subunit kd on gene expression programs. Some functions of esBAF appear to require functionality and cooperation of all subunits and are, therefore, altered regardless of which core member is impaired in function (i.a. regulation of *Cell Cycle* and *Metabolism*). Other functions, however, seem to be more dependent on particular subunits than on the faultless cooperation of all esBAF subunits. For instance, Smarcb1 is crucial for regulation of *Nervous System*-related genes, resulting in a more pronounced deregulation of those genes after Smarcb1 knockdown than after Smarca4 knockdown.

### 3.3. Upon Smarcb1 kd, Smarca4 Binding and Histone Marks Change in Gene Regions Connected to Nervous System, Cell Organisation and Adhesion

**Figure 3:**
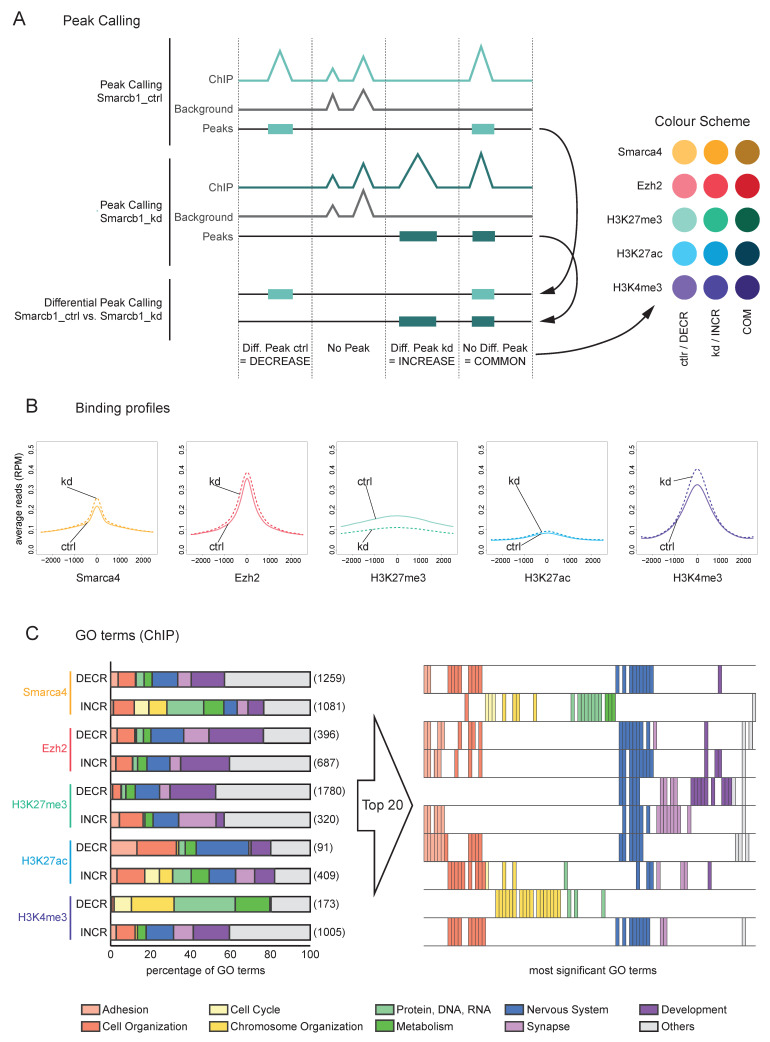
Smarcb1 loss causes binding changes in regions related to *Adhesion*, *Cell Organisation*, *Nervous System* and *Development* (**A**) Schematic overview of ChIP-seq peak calling. First, peaks have been called by comparing the antibody sample (“ChIP”) to their corresponding IgG control (“Background”). For evaluation of changes in binding, differential peak calling was performed. ChIP-seq results of control and *Smarcb1*-kd cells (after 72 h) were compared, resulting in the definition of three groups: DECREASE (more binding in the control sample), INCREASE (more binding in the *Smarcb1*-kd sample) and COMMON (no significant difference in binding). (**B**) LOESS-smoothed average binding profiles for all antibodies of this study. Control samples are depicted in a lighter colour scheme and *Smarcb1*-kd samples in darker colours. (**C**) GO terms assigned to genes with either a DECREASE or an INCREASE in the respective antibody further classified into categories. On the left-hand side, a summary of these categories is given; on the right-hand site, only GO terms within the top 20 (ranked by *p* value) in at least one antibody are included. In parenthesis, the total number of GO terms are given.

Aiming to unravel the changes in BAF complex as well as PRC2 binding on a genome-wide level, we performed chromatin immunoprecipitation followed by sequencing (ChIP-seq) in control vs. Smarcb1 kd 81K1 cells at timepoint 72 h. To ensure a sufficient Smarcb1 kd and to control the specificity of antibodies used for ChIP-seq experiments, Western blot analyses have been performed on protein lysates of cells later used for ChIP-seq (Supplementary Figure S3B). In addition, ChIP-qPCR has been used on various genes to validate the ChIP-seq experiment (selection displayed in Supplementary Figure S3C). Peak calling was performed as follows: first, peaks were called individually against the input sample (referred to as ctrl or kd). Second, differential peak calling was performed comparing peaks in the control vs. the Smarcb1-kd sample. Peaks that were more prominent in the control sample were referred to as DECREASE, peaks more prominent in the Smarcb1 kd sample as INCREASE and peaks present in both conditions were called COMMON (Figure 3A). Our analysis focuses on binding events within gene regions, which were defined as spreading from 1 kb upstream of the transcription start site (TSS) to the transcription end site (TES).

The peak profiles exhibit anticipated differences in factor binding. While Smarca4, Ezh2 and H3K4me3 display narrow peaks, H3K27me3 and H3K27ac are expectedly marked by broad peaks with lower average reads (Figure 3B). Smarca4 (21,523 peaks in control, 21,864 peaks in doxycycline-treated sample) and H3K4me3 (18,600 and 20,355, respectively) yielded the highest number of called individual peaks (Supplementary Figure S3A). Given the permissive nature of ESC chromatin [71], the high abundance of these marks is of no surprise. Binding of Smarca4 and Ezh2 as well as histone marks are not gained (or lost) on a genome-wide level, but rather at individual genomic sites. The latter is in line with the Western blots we performed (see above). However, the analysis of genomic sites with a DECREASE or INCREASE in binding events revealed differences between the factors. Surprisingly, Smarca4 binding was more frequently INCREASED than DECREASED, hinting towards a possible retargeting of the complex rather than a plain loss of the complex’s binding and function after Smarcb1 kd. Further, activating histone marks H3K27ac and H3K4me3 showed a similar pattern. Vice versa, inhibitory marks Ezh2 and H3K27me3 predominantly display a DECREASE in binding (Supplementary Figure S3A). This, similar to the findings of other working groups before, challenges the conception of esBAF and Ezh2 as solely antagonistic complexes.

To assess the biological implications of differentially bound genes, we performed GO term analyses individually per antibody (Figure 3C, more detailed display of GO terms in Supplementary Figure S4). Interestingly, summarising GO categories of different ChIP-seq samples mirror each other: changes in binding of all antibodies occur in gene regions connected to *Adhesion* and *Cell Organisation* (e.g., “biological adhesion” or “cell morphogenesis”) as well as *Nervous System* and *Synapse* (e.g., “generation of neurons” or “neurogenesis”). This finding supports and explains our findings in the gene expression analysis. As mentioned above, many differentially expressed genes are connected to the exact categories and GO terms that can also be identified when analysing changes in antibody binding after Smarcb1 kd. At this point, we conclude that Smarcb1 loss results in the alteration of chromatin structure especially in those regions where *Adhesion*-, *Cell Organisation*- and *Nervous System*-related genes are encoded, resulting in the deregulation of their expression.

However, genes with a Smarca4 INCREASE differ remarkably from most of the other datasets. They are associated with the previously named categories to a much lesser extent but are, in contrast, part of terms such as “chromosome organization”, “DNA metabolic process” and “cell cycle”. Likewise, genes with an H3K4me3 DECREASE are dominated by terms such as “chromatin organization”, “histone modification” or “DNA conformation change” (Figure 3C). Interestingly, Smarca4 INCREASE does not only differ from Smarca4 DECREASE regarding GO-term analysis, but also in the possible underlying mechanism. For Smarca4 DECREASE, it is possible to hypothesise that Smarcb1 loss causes a conformational and functional change of esBAF, resulting in the loss of contact to the DNA and other transcription factors and, therefore, to changes in chromatin conformation and ultimately in gene expression. For Smarca4 INCREASE, this explanation is implausible.

### 3.4. A Subset of Downregulated Genes with Smarca4 INCREASE after Smarcb1 kd Show an Unexpected Binding Pattern

**Figure 4:**
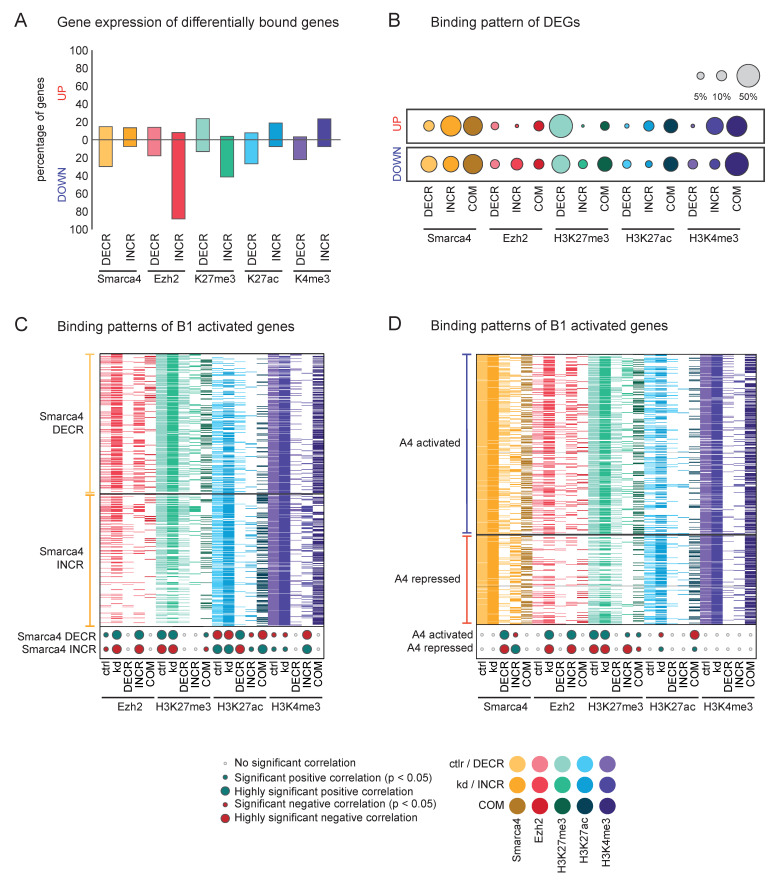
ChIP-seq defines two different groups of downregulated genes being separated by their antibody binding patterns (**A**) Bar chart displaying merged information of ChIP-seq and RNA-seq data. For analysis, all genes were included fulfilling two conditions: (**I**) DECREASE (DECR) or INCREASE (INCR) in binding of the respective antibody and (**II**) existing RNA-seq data (excluding genes with *p* value > 0.05 and abs. log2FC > 0.58). Relative amounts were calculated separately for each condition by dividing number of genes with differential binding of the respective antibody and up- (log2FC > 0.58) or down- (log2FC < −0.58) regulation by all genes, fulfilling (**I**) and (**II**). For (**B**), these were divided by all upregulated or downregulated genes, respectively (independent of antibody binding). (**C**) Heatmap showing antibody binding patterns of genes fulfilling the following criteria: (**I**) downregulated after *Smarcb1* kd (Smarcb1-activated) and (**II**) Smarca4 DECREASE or INCREASE. Depending on Smarca4 binding, two groups were formed whose antibody binding patterns were compared using permutation tests (results below the heatmap). Aquamarine circles represent a positive correlation (genes are more likely to be bound by the antibody of interest if they are in the tested group) while red circles represent a negative correlation (genes are less likely to be bound by the antibody of interest if they are in the tested group). For a more detailed explanation of significance levels, see Materials and Methods. (**D**) Heatmap displaying antibody binding patterns of genes (**I**) downregulated after *Smarcb1* kd (=Smarcb1-activated) and (**II**) changed expression after *Smarca4* kd (data obtained by Ho et al. [34]). Depending on expression after *Smarca4* kd, two groups were formed whose antibody binding patterns were compared using permutation tests (results below the heatmap).

Changes in chromatin binding of Smarca4 and Ezh2, as well as changes in binding patterns of histone marks, are connected to alterations in gene expression. To examine the correlation between factor occupancy and gene expression in more detail, we integrated the two datasets (Figure 4A). The inhibitory mark H3K27me3 primarily correlates with the expected changes: a DECREASE is connected to an upregulation of bound genes; an INCREASE is connected to a downregulation. Vice versa, a DECREASE in the activating histone marks H3K27ac and H3K4me3 causes a downregulation, while an INCREASE is connected to an upregulation. Ezh2 INCREASE is, as expected, associated with downregulation. Surprisingly, Ezh2 DECREASE is not always connected to an upregulation of affected genes. A Smarca4 DECREASE results in a downregulation, a Smarca4 INCREASE in an upregulation of corresponding genes (Figure 4A).

Focusing on RNA-seq results, upregulated genes are associated with a Smarca4 INCREASE, Ezh2 DECREASE (more than INCREASE), H3K27me3 DECREASE, H3K27ac INCREASE and H3K4me3 INCREASE. This antibody-binding pattern (DECREASE in inhibitory factors, INCREASE in activating factors) is expected for upregulated genes. However, downregulated genes show a similar amount of genes with Smarca4 DECREASE and INCREASE and of genes with Ezh2 DECREASE or INCREASE, respectively. Furthermore, they display a high number of genes with a H3K27me3 DECREASE (but also markedly more with a H3K27me3 INCREASE than upregulated genes) (Figure 4B).

We aimed to understand the differences between downregulated genes with a Smarca4 DECREASE and those with a Smarca4 INCREASE. For this, we separated these genes into two groups that were compared in a heatmap and a permutation analysis (Figure 4C). These two groups not only differ in their Smarca4 binding, but show different binding patterns of the other examined factors as well: downregulated genes with a Smarca4 DECREASE (upper part of the heatmap) display a binding pattern expected for downregulated genes: a positive correlation with Ezh2 binding, a more frequent Ezh2 INCREASE, a more prominent H3K27me3 binding, an H3K27ac DECREASE as well as a less prominent H3K4me3 binding. Vice versa, downregulated genes with a Smarca4 INCREASE are marked by a binding pattern opposed to the described one (Figure 4C). This second group challenges the consequences of a *Smarcb1* loss described in literature (e.g., in tumour cells), loss of BAF binding and repression of genes via PRC2 activity [72], and in this way suggest the existence of additional mechanisms by which BAF complexes control gene repression.

As we have already stated regarding the striking differences between a *Smarcb1* versus *Smarca4* knockdown, we wanted to correlate the contrasts in binding patterns described above to the changes in gene expression observed after *Smarca4* knockdown. Indeed, we could observe a distinct binding pattern when comparing B1-/A4-activated genes (downregulated after Smarcb1 kd and Smarca4 kd) with B1-activated, not A4-activated genes (downregulated after Smarcb1 kd but not after Smarca4 kd) (Figure 4D). The first group is more likely to show a *Smarca4* DECREASE as well as the described binding pattern of Ezh2 INCREASE, H3K27me3 INCREASE and less H3K27ac binding. *Vice versa*, the second group is more likely to display a Smarca4 INCREASE with the corresponding binding pattern of less Ezh2, less H3K27me3 and more H3K27ac binding (Figure 4D). In conclusion, genes that are downregulated after Smarcb1 kd but not after Smarca4 kd show a binding pattern that is unexpected for downregulated genes: more Smarca4 INCREASE, less Ezh2 INCREASE, less H3K27me3 INCREASE, more H3K27ac binding.

When focusing on upregulated genes, most of them show a Smarca4 INCREASE and only a minority show a Smarca4 DECREASE. When comparing these groups, there are no marked differences and the comparison with the dataset after Smarcb1 kd is also insignificant (Supplementary Figure S5).

### 3.5. Smarcb1 Loss Results in Changes in Enhancer Occupancy, Particularly in Regions Regulating Nervous System-, Cell Organisation-, Adhesion- and Chromosome Organisation-Related Genes

**Figure 5:**
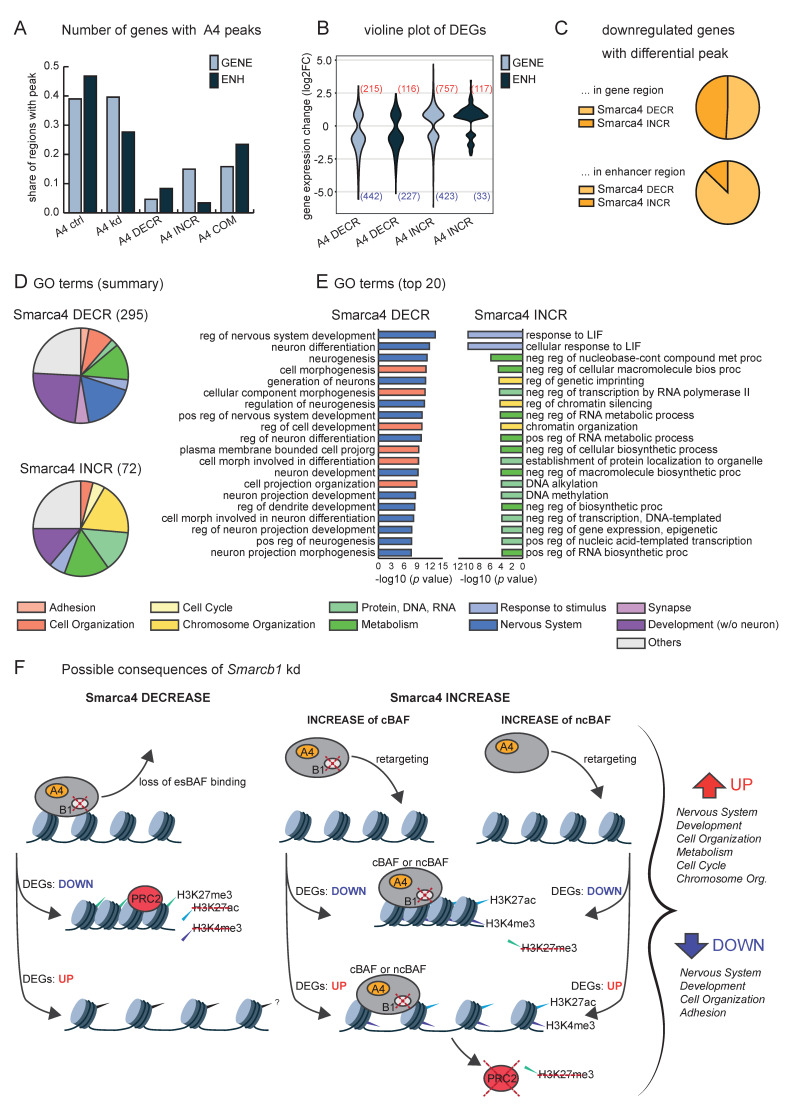
*Smarcb1* knockdown impacts BAF complex binding in enhancer regions (**A**) Comparison of Smarca4 binding of regions defined either as gene regions (1 kbp upstream of TSS to TES) or as enhancer regions (as defined by Shen et al. [68]). (**B**) Correlation of enhancer regions to the nearest gene (max. distance of 100,000 bp). Gene expression of those genes that were significantly deregulated (*p* < 0.05, abs. log2FC > 0.58) are displayed in violine plots and their number is given in parenthesis. (**C**) Pie charts displaying the amount of genes with Smarca4 DECREASE or INCREASE relative to all downregulated genes with a change in Smarca4 binding. (**D**,**E**) Biological process related GO terms that were assigned to genes with differential Smarca4 binding in their enhancer region. Genes with Smarca4 DECREASE or INCREASE were analysed separately using ToppGene and terms were separated into categories (as used for other GO-term analyses). Pie charts give a summary of these categories, bar charts only include GO terms within the top 20 (ranked by *p* value). In parenthesis, total numbers of GO terms are given. (**F**) Summary of possible consequences of *Smarcb1* kd for BAF-complex function. In part, mechanisms by which changes in gene expression occur remain unclear (especially concerning upregulated genes with Smarca4 DECREASE and downregulated genes with Smarca4 INCREASE without Ezh2 INCREASE).

BAF-complex occupancy is typical for enhancers and super enhancers [73,74,75]. Therefore, we investigated whether a Smarca4 association with known enhancer regions could be linked to the observed gene expression changes in Smarcb1-kd cells. We defined enhancer regions with the help of a dataset published by Shen et al. [68] in murine ESC. About 47% of enhancers were bound by Smarca4 in control cells, but only about 27% in Smarcb1-kd ESC. Consequently, the number of enhancers with a Smarca4 DECREASE exceeds the number of enhancers with a Smarca4 INCREASE, while it is vice versa in gene regions (Figure 5A).

Enhancers were further linked to the nearest gene (maximum 100,000 kb) (Figure 5B). While gene and enhancer regions with Smarca4 DECREASE barely differ in their effect on gene expression (about 1/3 are upregulated and about 2/3 downregulated), a Smarca4 INCREASE differs in its consequences depending on if it occurs in gene or enhancer regions. Only about 64% of genes with a Smarca4 INCREASE in their gene region are upregulated, but 78% of genes with a Smarca4 INCREASE in their enhancer region (Figure 5B). Moreover, downregulated genes are less likely to show an INCREASE in Smarca4 at linked enhancers than within genes: of the downregulated genes with a change in Smarca4 binding in their gene region, about 50% show a Smarca4 DECREASE and 50% a Smarca4 INCREASE. In contrast, downregulated genes with a change in Smarca4 binding in their enhancer region are much more likely to show a Smarca4 DECREASE (87%) than a Smarca4 INCREASE (Figure 5C). Possibly, this illuminates two distinct modes of operation of the esBAF complex. (I) When binding to enhancer regions, it is mainly restricted to its activating role, making enhancer regions accessible and allowing the expression of genes controlled by these enhancers. (II) When directly binding to gene regions, it can be activating and repressive, in part by the pre-described mechanisms of direct PRC2 (and PRC1) antagonism, in part by collaborating with PRC2 and possibly in part by other repressive mechanisms independent of PRC2.

In addition, we investigated the biological function of genes with changes in Smarca4 enhancer occupancy (Figure 5D,E). GO terms connected to genes with a Smarca4 DECREASE in their enhancer regions are dominated by terms included in the category *Nervous System* (e.g., “neurogenesis”) as well as *Cell Organisation* (e.g., “Cell Projection Organization”). In contrast, those with a Smarca4 INCREASE in their enhancer region are more dominated by terms connected to nucleic acid metabolism, other nucleic acid-related terms such as “negative regulation of transcription, DNA-templated” and *Chromosome Organisation*-related terms (Figure 5D,E). Similar to the analysis of gene expression data and of antibody binding after Smarcb1 loss, it becomes evident that *Nervous System*- as well as *Cell Organisation*- and *Adhesion*-related genes are severely influenced by the loss of this esBAF subunit. Many of these genes are (I) deregulated in their expression and (II) show a change in Smarca4 (and, therefore, esBAF) binding in gene regions as well as enhancer regions.

## 4. Discussion

Our data highlight the pivotal role of Smarcb1 in the regulation of nervous system development and function. Not only are genes that are differentially expressed after Smarcb1 kd connected to these biological functions by GO-term analysis, but those genes also show differences in antibody binding in ChIP-seq, providing a plausible explanation for changes in expression. Further, the regulation of nervous system-related genes appears to be a predominant function of *Smarcb1*, as the knockdown of core esBAF subunit *Smarca4* only has limited impact on them. Especially genes uniquely deregulated after Smarca4 kd are connected to nervous system-related processes to a much lesser extent. We conclude that *Smarcb1* expression and function is indispensable for neurogenesis, neuron differentiation and, therefore, brain development. In fact, data from our group demonstrate that *Smarcb1* loss in mice results in severe brain developmental defects when occurring at an early timepoint during embryonic development [76]. In addition, the importance of SMARCB1 for neural development has been shown in human iPSCs [77]. Importantly, *Smarcb1* alterations are linked to neurodevelopmental disorders and tumours of the developing brain such as atypical teratoid rhabdoid tumours (AT/RT). In both cases, early alterations in *Smarcb1* function (rather than in the mature brain) are most likely to initiate disease development as, e.g., children with Coffin-Siris Syndrome are symptomatic at birth and AT/RT exclusively develop in early childhood [78,79].

In parallel to describing transcriptional changes after Smarcb1 kd, we aimed to understand underlying mechanisms. It has already been described that a loss of *Smarcb1* results in a loss of the BAF complex’s ability to evict PRC2 from the chromatin and, in this way, damages the antagonistic relation between these complexes [36]. Additionally, their antagonistic relation is the reason why BAF-complex function has been perceived as activating genes. This notion has already been challenged in ESC by Ho et al. [34] but also by Weber et al. [35], who described an agonistic function of BAF and PRC2 when supressing *Hox* cluster gene expression in mESC. Our work supports these findings and shows that changes in gene expression after Smarcb1 kd are not dominated by the downregulation of genes, as would be expected if the BAF complex was solely activating.

In our analysis, we discriminated between genes with a Smarca4 DECREASE and INCREASE. These two groups do not only differ regarding gene functions (as revealed by our GO-term analysis) but also in the possible underlying mechanism (Figure 5F). For Smarca4 DECREASE, it is possible to hypothesise that Smarcb1 loss causes a conformational and functional change of esBAF, resulting in the loss of contact to the DNA and other transcription factors and, consequently, to changes in chromatin conformation and ultimately gene expression. For Smarca4 INCREASE, it seems likely that the esBAF complex is not only altered in its function at canonical BAF-regulated sites but is retargeted to gene regions where it usually does not bind. Consequently, there are two possible explanations for the upregulation of genes after Smarcb1 kd: (I) a repressive esBAF complex is impaired in its function and fails to repress this gene or (II) an activating esBAF complex was retargeted to a gene that is not esBAF-activated in Smarcb1-positive mESC. The fact that GO terms such as “cell cycle” can be found in both datasets, upregulated genes as well as genes with Smarca4 INCREASE, further supports that (II) should be considered when aiming to understand alterations in esBAF functions after *Smarcb1* loss.

Furthermore, these two groups (Smarca4 DECREASE vs. INCREASE) can also be applied to downregulated genes. Again, two distinct mechanisms by which gene expression is changed seem plausible: (I) an activating esBAF complex is impaired in its function and (II) a repressive esBAF complex is retargeted to non-canonical target sites. Interestingly, these groups also differ in their antibody-binding patterns. As expected, when having the BAF–PRC2 antagonism in mind, genes with a Smarca4 DECREASE show an INCREASE in Ezh2, a more prominent H3K27me3 binding and a DECREASE in activating histone marks. In contrast, genes with Smarca4 INCREASE present the opposite changes. As a result, this retargeted BAF complex could initiate gene silencing by other means than PRC2 action, for example, by interaction with other inhibitory complexes.

In addition to differences between Smarca4 binding of down- and upregulated genes, there are also differences between gene and enhancer regions. As described, in enhancer regions, a Smarca4 DECREASE is much more frequent than a Smarca4 INCREASE (while it is vice versa in gene regions).

It is difficult to explain why the kd of Smarcb1 should result in a retargeting of the BAF complex rather than in a loss of binding and why this seems to occur more frequently in gene regions. However, a retargeting becomes evident in our data and has also been shown in data published on synovial sarcoma, a cancer entity in which BAF complexes contain an SS18-SSX fusion protein but also lose SMARCB1 in their complexes [80]. Recent research challenged the perception of Smarcb1 as an essential, non-replaceable subunit of the BAF complex. Amongst others, Alpsoy and Dykhuizen [29] described that the ncBAF complex, in contrast to all BAF complexes described before, does not contain Smarcb1 as a subunit. Even if it differs in subunit composition and in its function and preferred binding sites, the ncBAF has been shown to contribute to the regulation of mESC pluripotency [30]. Thus, it is possible that sites with a Smarca4 INCREASE are sites where the ncBAF complex binds and gains more importance in a cell where the cBAF complex is impaired in its functions as its core subunit Smarcb1 is lost. Possibly, this ncBAF complex has a limited affinity to enhancer regions [30], explaining why we do not see the same amount of Smarca4 INCREASE in these regions. Interestingly, in *Smarcb1* deficient cancer entities, ncBAF specific subunit BRD9 was identified as being essential for tumour cell proliferation, further supporting the idea that the ncBAF ensures cells’ survival by taking over cBAF functions [81,82].

## 5. Conclusions

In conclusion, Smarcb1 function is essential for the accurate regulation and expression of *Nervous System*-related genes. After Smarcb1 knockdown, antibody binding patterns of these regions and their corresponding enhancers are altered, resulting in a change of expression. Genome-wide, we observe both a DECREASE and an INCREASE in esBAF binding, hinting towards different mechanisms and causing gene deregulation. While parts of the observed gene expression changes can be explained by an esBAF–PRC2 antagonism, others appear to be PRC2-independent. Possibly, the takeover of the ncBAF complex is responsible for a subset of alterations we observed, especially regarding sites with a Smarca4 INCREASE after Smarcb1 kd (Figure 5F). A more detailed understanding of these mechanisms, e.g., by ncBAF specific ChIP-seq, could also result in more profound knowledge about the pathogenesis of diseases of the nervous system occurring in early development, such as Coffin-Siris Syndrome or AT/RT.

## Data Availability

RNA-seq and ChIP-seq data have been deposited in NCBI’s Gene Expression Omnibus and are accessible through GEO Series accession numbers GSE186669.

## Supplementary Materials

The following supporting information can be downloaded at: https://www.mdpi.com/article/10.3390/cells11081354/s1, Figure S1 (related to Figure 1): Smarcb1 loss has little impact on Ezh2 expression and histone marks but impairs mESC proliferation, Figure S2 (related to Figure 1): In-detail analysis of gene expression changes after Smarcb1 kd and Smarca4 kd, Figure S3 (related to Figure 3): Validation of ChIP-seq data, Figure S4 (related to Figure 3): Detailed analysis of GO terms connected to genes with changed antibody binding after Smarcb1 kd, Figure S5 (related to Figure 4): Genes upregulated after Smarcb1 knockdown share their antibody binding pattern, Table S1: Log2FC thresholds for comparison of Smarca4 versus Smarcb1 kd, Table S2: Lysis buffers used for chromatin extraction, Table S3: Oligonucleotides‘ sequences, Table S4: shRNA sequences, Table S5 Antibodies used in this study, Table S6 Chemicals used in this study.

## Abbreviations

A4	Smarca4
abs	absolute
B1	Smarcb1
bios	biosynthetic
cBAF	Canonical BAF complex
COM	COMMON
ctrl	control
DE(G)	differentially expressed (gene)
DECR	DECREASE
dox	doxycycline
ENH	enhancer region
esBAF	ESC-specific BAF complex
GENE	gene region
GO	Gene ontology
HMT	histone methyltransferase
INCR	INCREASE
K27ac	H3K27ac
K27me3	H3K27me3
K4me3	H3K4me3
kd	knockdown
LIF	leukaemia inhibitory factor
(m)ESC	(murine) embryonic stem cell
morph	morphogenesis
ncBAF	non-canonical BAF complex
neg	negative
PBAF	Polybromo-associated BAF complex
pos	positive
PRC1	Polycomb Repressive Complex 1
PRC2	Polycomb Repressive Complex 2
proc	process
prot	protein
rec	receptor
reg	regulation
RMCE	recombinase-mediated cassette exchange
sign	signalling
TES	transcription end site
TSS	transcription start site
