# Supplementary material for: Smarcb1 Loss Results in a Deregulation of esBAF Binding and Impacts the Expression of Neurodevelopmental Genes
Source: Cells. 2022 Apr 15;11(8):1354. doi: 10.3390/cells11081354 (PMC9027123; doi:10.3390/cells11081354)
Supplement: Supplementary file 1 [file cells-11-01354-s001.zip › cells-1607031-supplementary.pdf]

SUPPLEMENTARY FIGURES

Supplementary Figure S1

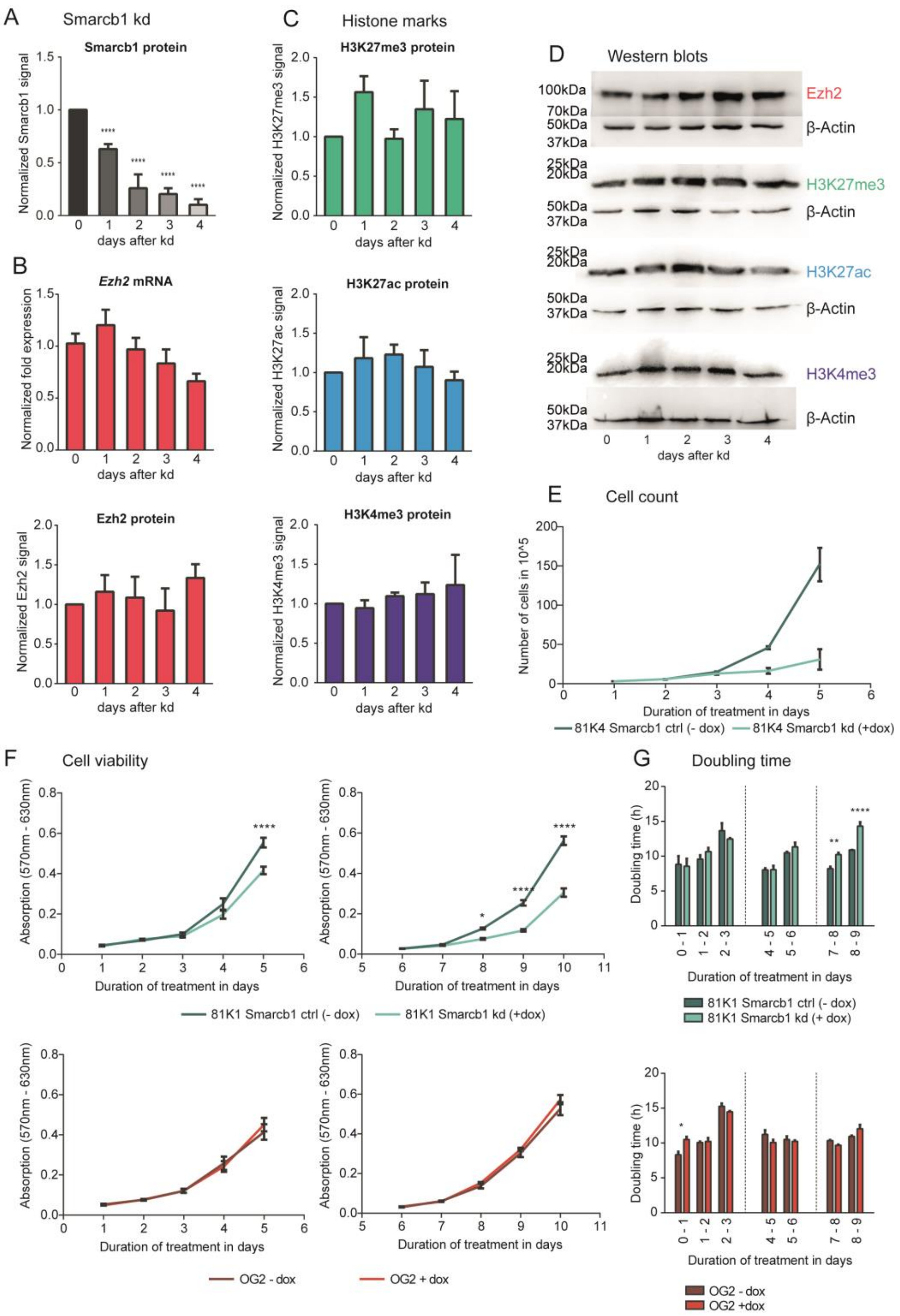

Supplementary Figure S1. (related to Figure 1): Smarcb1 loss has little impact on *Ezh2* expression and histone marks but impairs mESC proliferation (A) Levels of Smarcb1 protein in Western blot semi-quantitatively analysed using Image J including n = 3 biological replicates. (B) *Ezh2* expression in RT-qPCR (n = 3 biological replicates in technical triplicates) and Ezh2 signal in Western blot semi-quantitatively analysed using Image J (n = 3 biological replicates). (C)

Western blots of histone marks H3K27me3, H3K27ac and H3K4me3 semi-quantitatively analysed using Image J. Per antibody, experiments were carried out in n = 3 biological replicates. (D) Representative Western blots for all antibodies presented in (B) and (C). (E) Manual counting experiment of 81K4 ESC clone in n = 3 biological replicates. (F) MTT assays performed in n = 3 biological replicates per condition with 81K1 ESC with and without kd of *Smarcb1*. As control cell line, OG2 ESC were tested under equal conditions. Cells treated for 1 to 5 days and cells treated for 6 to 10 days (pre-treated before seeding for MTT assay) were examined in two different experiments to ensure optimal growth conditions. (G) Doubling time determined by manual counting in n = 3 independent experiments. In (A) – (C) and (E) – (G), error bars indicate SEM, * = $p < 0.05$, ** = $p < 0.01$, *** = $p < 0.001$, **** = $p < 0.0001$.

Supplementary Figure S2

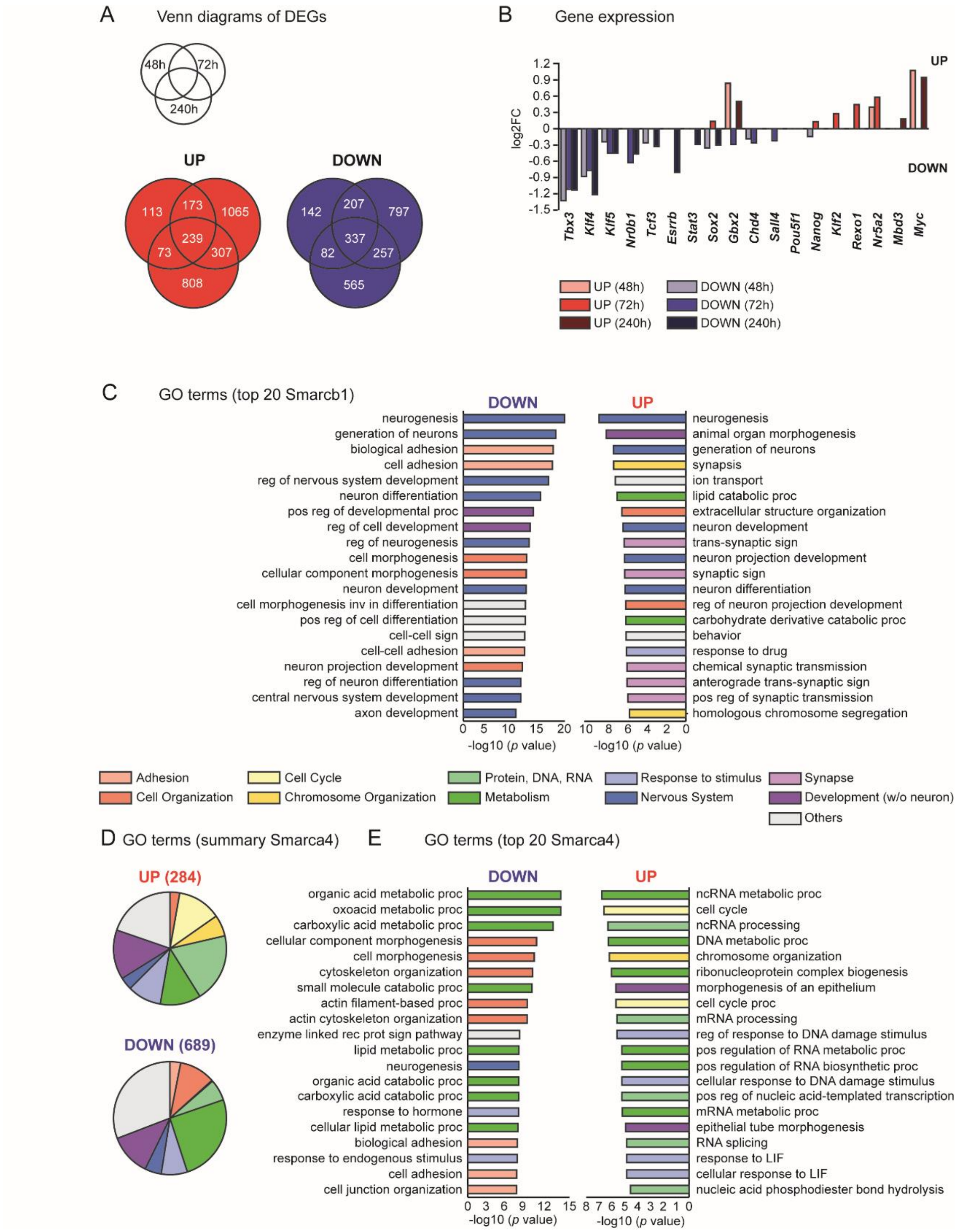

Supplementary Figure S2. (related to Figure 1): In-detail analysis of gene expression changes after *Smarcb1* kd and *Smarca4* kd (A) Venn diagrams of DEG after *Smarcb1* kd at three different time points (48 h, 72 h, 240 h). Only genes with p value < 0.05 and abs. log2FC > 0.58 were considered. (B) Expression changes of genes connected to naïve pluripotency in ESC that were described by Dunn et al. [4] 48 h, 72 h and 240 h after *Smarcb1* kd. Only expression of genes with $p < 0.05$ is displayed. (C) Top 20 GO terms (ranked by p value) assigned to up- and downregulated genes after 72 h of *Smarcb1* kd. Colour code is based on categories as explained in Fig. 1E. (D and E) Biological process related GO terms that were assigned to genes up- or downregulated after

Smarca4 knockdown (data obtained from [34]). Genes were analysed separately using ToppGene and terms were separated into categories already used for *Smarcb1* knockdown related GO terms. Pie charts give a summary of these categories, bar charts only include GO terms within the top 20 (ranked by *p* value) In parenthesis, total numbers of GO terms are given.

Supplementary Figure S3

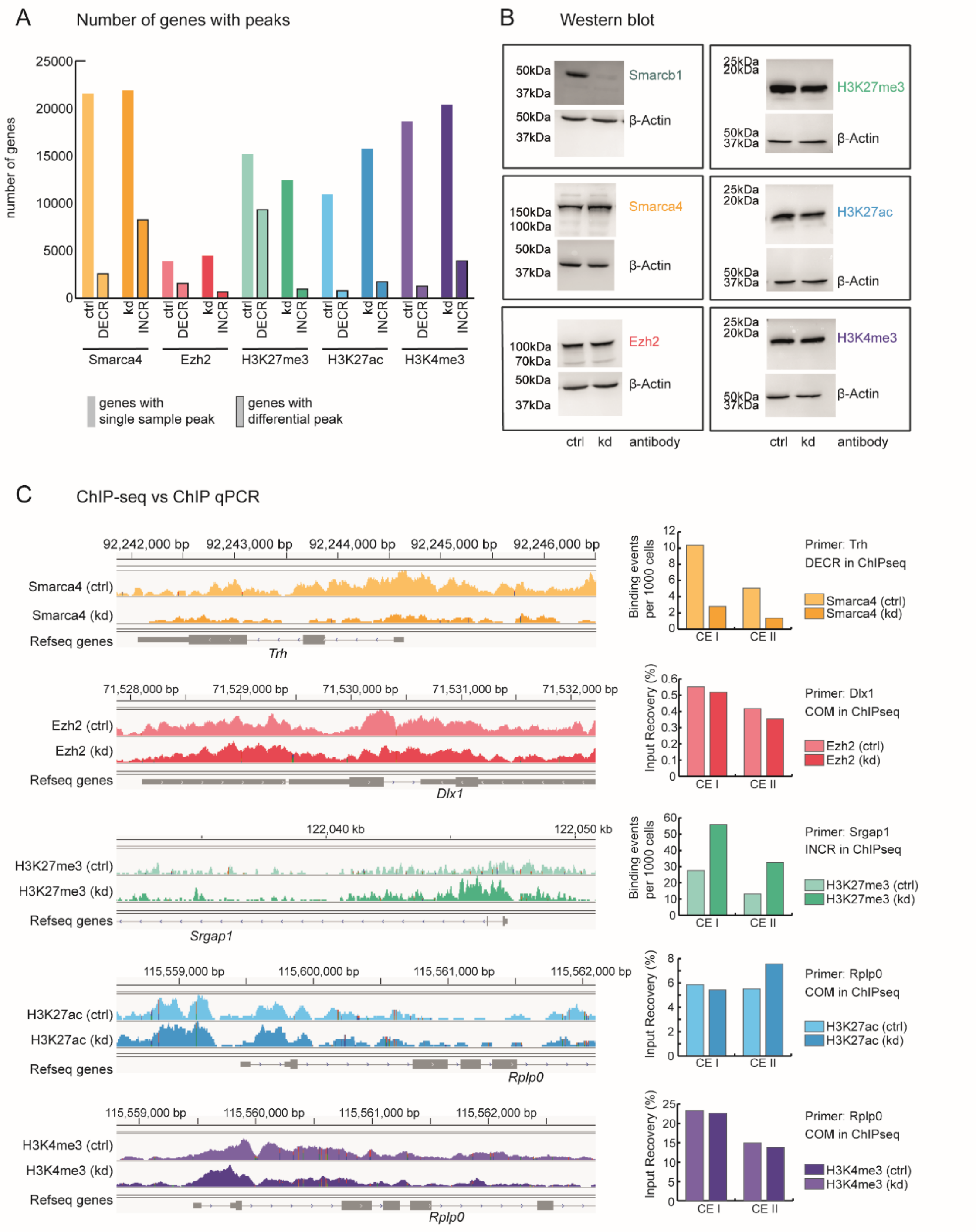

Supplementary Figure S3. (related to Figure 3): Validation of ChIP-seq data (A) Number of genes with peaks / DECREASE / INCREASE of examined factors and histone marks. For merging ChIP-seq data with genomic information, a gene region was defined to start 1 kbp upstream of TSS (Transcription start site) until TES (Transcription end site). (B) Representative Western blots performed with protein extracts of cells that were also used for ChIP-seq. (C) Validation of ChIP-seq experiments via ChIP-qPCR. Gene regions with antibody binding were chosen randomly and peak calling after ChIP-seq (left-hand) were compared with ChIP-qPCR results (right-hand). For a more detailed review on analysis of ChIP-qPCR, see materials and methods. ChIP-qPCR experiments were performed in n = 2 biological replicates.

Supplementary Figure S4

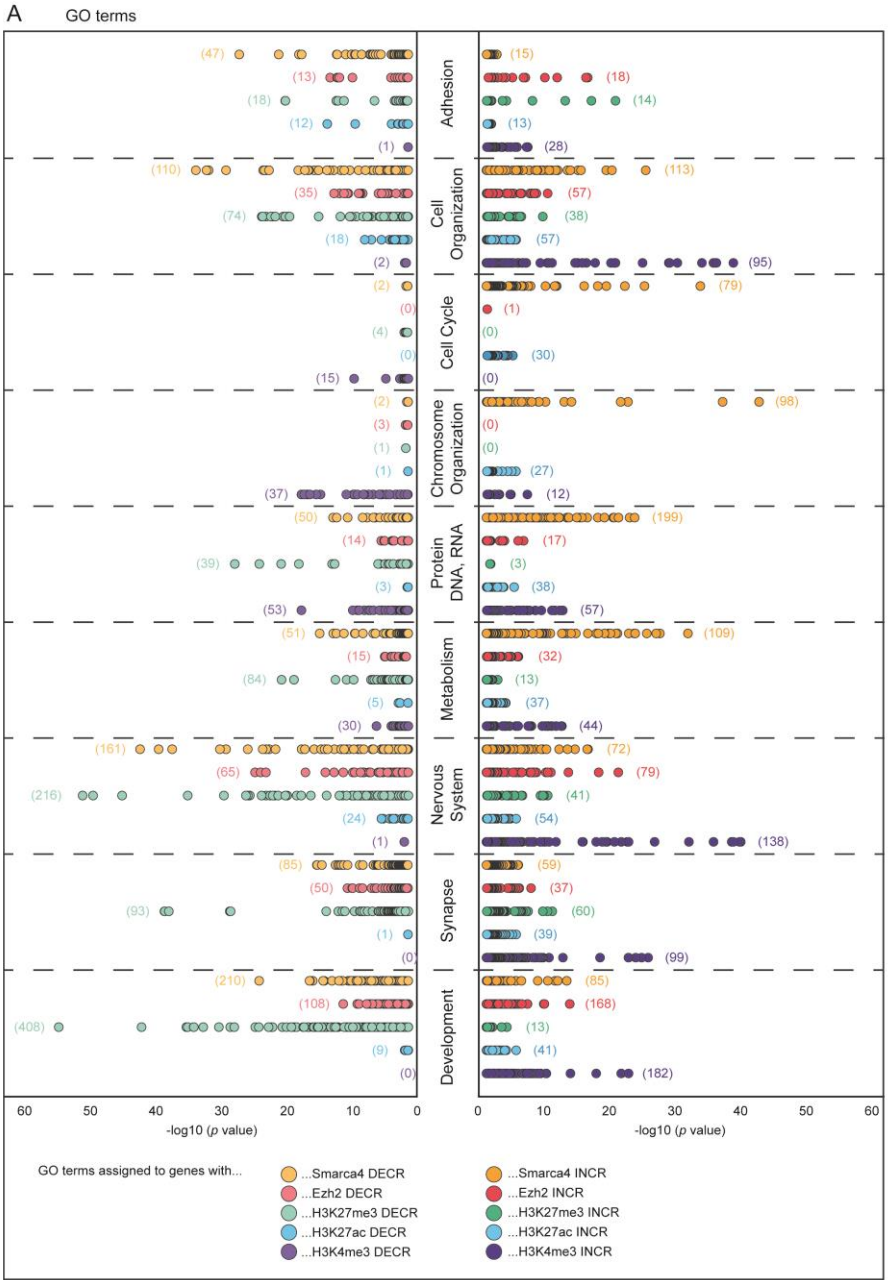

Supplementary Figure S4. (related to Figure 3): Detailed analysis of GO terms connected to genes with changed antibody binding after *Smarcb1* kd (A) GO terms being assigned to the indicated gene-sets (genes with changed antibody binding after *Smarcb1* knockdown). On the left-hand site, terms associated with genes showing a DECREASE of antibody binding (lighter colours) are represented, on the right-hand site, those with an INCREASE (darker colours.) Each dot represents one individual GO term, numbers in parentheses report the overall number of GO terms that have been assigned to the gene set and category of interest. Terms fitting into more than one category are depicted in all of these categories to prevent loss of information. This graph focuses on the most frequently occurring topics, therefore not including all GO terms that are presented in Figure 3C.

Supplementary Figure S5

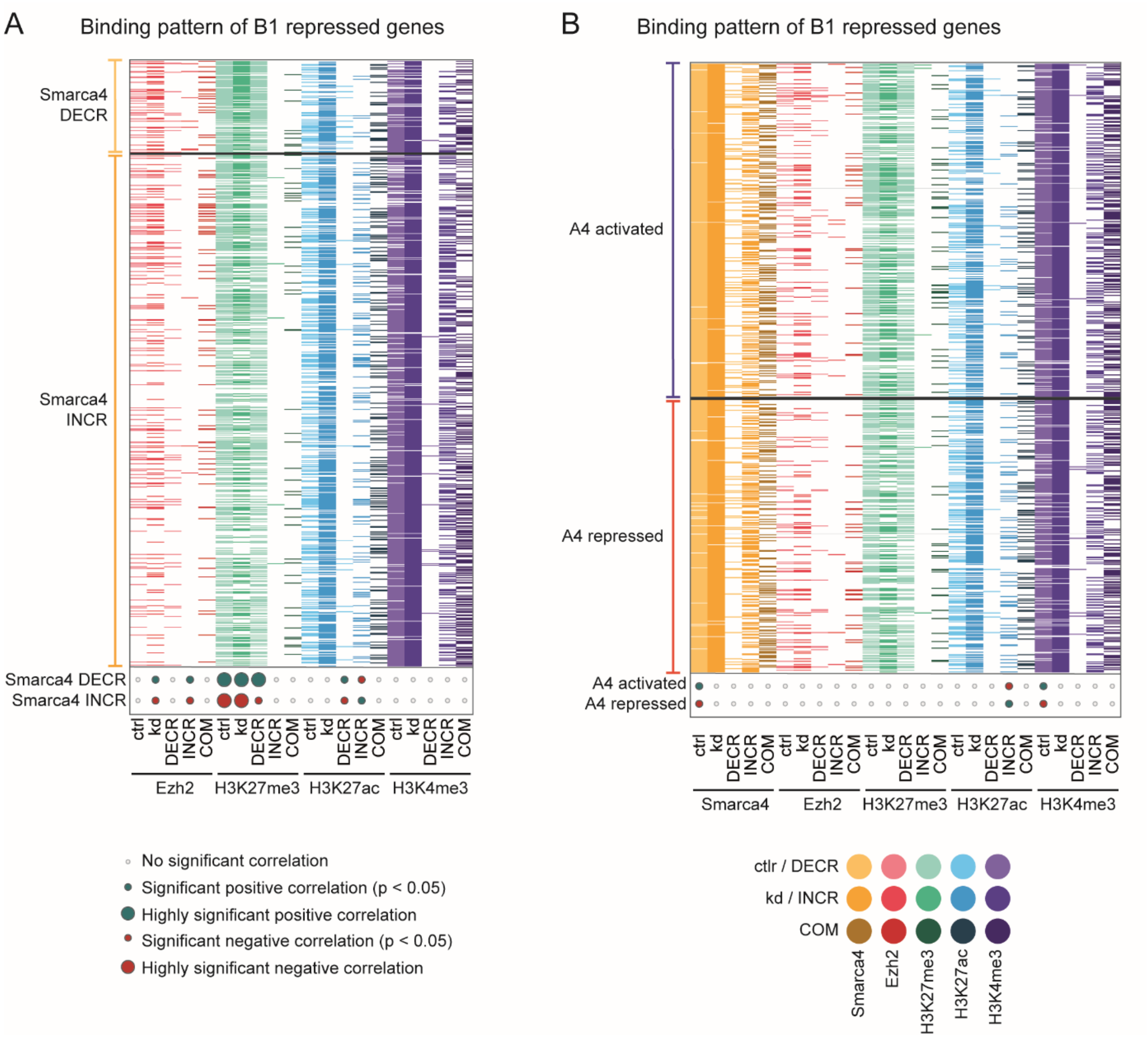

Supplementary Figure S5. (related to Figure 4): Genes upregulated after *Smarcb1* knockdown share their antibody binding pattern (A) Heatmap showing antibody binding patterns of genes fulfilling the following criteria: (I) upregulated after *Smarcb1* kd ($p < 0.05$, log2FC > 0.58) and (II) Smarca4 DECREASE or INCREASE. Depending on Smarca4 binding, two groups were formed whose antibody binding patterns were compared using permutation tests (results below the heatmap). Aquamarine circles represent a positive correlation (genes are more likely to be bound by the antibody of interest if they are in the tested group) while red circles represent a negative correlation (genes are less likely to be bound by the antibody of interest if they are in the tested group). For a more detailed explanation of significance levels, see materials and methods. (B) Heatmap displaying antibody binding patterns of genes (I) upregulated after *Smarcb1* kd ($p < 0.05$, abs. log2FC > 0.58) and (II) changed expression after *Smarca4* kd (data obtained from Ho et al. [34]). Depending on expression after *Smarca4* kd, two groups were formed whose antibody binding patterns were compared using permutation tests (results below the heatmap).

SUPPLEMENTARY TABLES

Supplementary Table S1: Log2FC thresholds for comparison of Smarca4 versus Smarcb1 kd

CATEGORY	DEFINITION

B1 / A4 repressed	Upregulated after Smarcb1 knockdown<br>→ p < 0.05, log2FC > 0<br>AND<br>Upregulated after Smarca4 knockdown<br>→ p < 0.05, log2FC > 0
B1 / A4 activated	Downregulated after Smarcb1 knockdown<br>→ p < 0.05, log2FC < 0<br>AND<br>Downregulated after Smarca4 knockdown<br>→ p < 0.05, log2FC < 0
B1 repressed / not A4 repressed	Upregulated after Smarcb1 knockdown<br>→ p < 0.05, log2FC > 0.58<br>AND<br>Not Upregulated after Smarca4 knockdown<br>→ either p > 0.05 or p < 0.05 and log2FC < 0
B1 activated / not A4 activated	Downregulated after Smarcb1 knockdown<br>→ p<0.05, log2FC< -0.58<br>AND<br>Not Downregulated after Smarca4 knockdown<br>→ either p >0.05 or p < 0.05 and log2FC > 0
A4 repressed / not B1 repressed	Upregulated after Smarca4 knockdown<br>→ p < 0.05, log2FC > 0.58<br>AND<br>Not Upregulated after Smarcb1 knockdown<br>→ either p > 0.05 or p < 0.05 and log2FC < 0
A4 activated / not B1 activated	Downregulated after Smarca4 knockdown<br>→ p < 0.05, log2FC < -0.58<br>AND<br>Not Downregulated after Smarcb1 knockdown<br>→ either > 0.05 or p < 0.05 and log2FC > 0

Supplementary Table S2: Lysis buffers used for chromatin extraction

INGREDIENTS	LB1	LB2	LB3
HEPES KOH<br>1 M, pH 7.4	50 mM	-	-

Tris HCl<br>1 M, pH 8.0	-	10 mM	10 mM
NaCl<br>5 M	140 mM	200 mM	140 mM
EDTA<br>0. 5 M, pH8.0	1 mM	1 mM	1 mM
EGTA<br>0.5 M, pH 8.0	0.5 mM	0.5 mM	0.5 mM
Glycerol Anhydrous	10 %	-	-
IGEPAL CA-630, 10 %	0.5 %	-	-
Triton X-100, 10 %	0.25 %	-	-
N-lauroyl-sarcosine, 10 %	-	-	1 %
Sodium deoxycholate	-	-	0.2 %

Supplementary Table S3: Oligonucleotides‘ sequences

**OLIGONUCLEOTIDES**	**FORWARD**	**REVERSE**
mSmarcb1	GAGGTGGGAAACTACCTGCG	CGCCAGAGTGAGGGGTATC
mRpl3	GGAAAGTGAAGAGCTTCCCTAAG	CTGTCAACTTCCCGGACGA
mEzh2	AATCAGAGTACATGCGACTGAGA	GCTGTATCCTTCGCTGTTTCC
mDlx1	ATGTCTCCTTCTCCCATGTCC	ACTGCACGGAACTGATGTAGG

mRplp0	GAATAAAATCTCTGCCCTGTGG	TACTCTCCCTTACTCTCCCACCT

Supplementary Table S4: shRNA sequences

**shRNA**	**Anti-*Smarcb1***
**Upper**	TCCCGAAGCTAATGACTCCTGAGATTTCAACAGAATCTCAGGAGTCATTAGCTTCTTTTT
**Lower**	CGCGTAAAAAGAAGCTAATGACTCCTGAGATTCTCTTGAAATCTCAGGAGTCATTAGCT

Supplementary Table S5: Antibodies uses in this study

**ANTIBODIES**	**SOURCE**	**IDENTIFIER**
**beta-actin mouse, monoclonal**	Santa Cruz Biotechnology	SC-47778
**α-Tubulin (B-7)**	Santa Cruz Biotechnology	sc-23948
**Smarca4 Rabbit, monoclonal**	Abcam	ab110641
**Brg1 (H-88)**	Santa Cruz Biotechnology	sc-10768
**Brg1 (N-15)**	Santa Cruz Biotechnology	sc-8749
**anti-Baf155**	Santa Cruz Biotechnology	sc-9746
**Baf155 (D7F8S)**	Cell Signaling Technology	9053
**EZH2 (D2C9) Rabbit, monoclonal**	Cell signalling	5246S
**H3K27ac**	Abcam	ab4729

**Rabbit, polyclonal**		
**H3K27me3**<br>**Rabbit, polyclonal**	Merck KGaA	07-449
**H3K4me3**<br>**Rabbit, polyclonal**	Diagenode	pAb-003-050
**IgG**<br>**Rabbit, monoclonal**	Novusbio	NBP2-24891
**Peroxidase-conjugated anti-Mouse**<br>**Goat, polyclonal**	Jackson Immuno Research	115-035-044
**Peroxidase-conjugated anti-rabbit**<br>**Goat, polyclonal**	Jackson Immuno Research	111-035-045
**Smarcb1**<br>**mouse, monoclonal**	BD transduction laboratories	612110
**Smarcb1 (Y-7)**	Santa Cruz Biotechnology	sc-101161

Supplementary Table 6: Chemicals used in this study

CHEMICALS	SOURCE	IDENTIFIER
**Absolute pure ethanol (75 %)**	AppliChem	# A4230-1000PE
**Acrylamide (30 %) / Bis Solution**	Bio-Rad Life Science	1610156
**A/G PLUS agarose beads**	Santa Cruz Biotechnology	sc-2003

**BSA (bovine serum albumin, molecular biology grade, 20 mg/ml)**	New England BioLabs	B9000S
**BSA (bovine serum albumin, fraction V, 7.5 %)**	Fisher Scientific	15260037
**Chloroform**	Sigma-Aldrich Chemie GmbH	288306
**cOmplete^{TM}**	Sigma-Aldrich Chemie GmbH	11697498001
**Coomassie Brilliant Blue G-250**	Bio-Rad Life Science	# 1610406
**DEPC water**	Invitrogen	AM9915G
**DMEM Glutamax**	Invitrogen	31966021
**Doxycycline**	Sigma-Aldrich Chemie GmbH	D9891-1G
**EDTA**	Carl Roth GmbH & CoKG	8043.2
**EGTA**	Sigma-Aldrich Chemie GmbH	E4378
**FBS superior**	Biochrom	S0615
**Formaldehyde**	Sigma-Aldrich Chemie GmbH	F8775

**Glycine**	Carl Roth GmbH & CoKG	3790.2
**Gylcerol**	AppliChem GmbH	A1123,2500
**HCl**	Honeywell Fluka™	71763
**HEPES KOH**	Sigma-Aldrich Chemie GmbH	H3375
**IGEPAL CA-630**	Sigma-Aldrich Chemie GmbH	18896
**IgG Dynabeads™**	Novex by Life Technologies	10004D
**Isopropanol**	SAV Liquid Production GmbH	ISOP-5000-100-1
**Laemmli Sample Buffer (4 X)**	Bio-Rad Life Science	1610747
**LiCl**	Sigma-Aldrich Chemie GmbH	62476
**MEM Non-essential amino acids**	Sigma-Aldrich Chemie GmbH	M7145
**Methanol**	Carl Roth GmbH & CoKG	T145.2
**MTT reagent**	Merck KGaA	CT01
**Murine embryonic stem cell leukaemia inhibitory factor (ESLIF)**	PolyGene Transgenetics	PG-A1140-0100

**NaCl**	Carl Roth GmbH & CoKG	3957.2
**NaDOC**	Sigma-Aldrich Chemie GmbH	D6750
**N-lauroyl-sarcosine**	Sigma-Aldrich Chemie GmbH	L9150
**Power SYBR Green PCR Master Mix**	Life Technologies	4367659
**Proteinase K**	Sigma-Aldrich Chemie GmbH	3115828001
**PVDF membranes**	BioRad Life Science	1620177
**RNase A**	Invitrogen	AM2272
**SDS**	Sigma-Aldrich Chemie GmbH	74255
**Triton X-100**	AppliChem GmbH	A4975,0500
**TRIzol®**	LifeTechnologies	15596018
**Trypsin 0.05 % EDTA**	Gibco by life technologies	25300054
**Western Blot ECL Pro Solution**	Perkin Elmer	NEL120001EA
**b-Mercaptoethanol**	Gibco by life technologies	31350-010

Supplementary Table S7: critical commercial kits

CRITICAL COMMERCIAL KITS	SOURCE	IDENTIFIER
**PrimeScript RT Reagent Kit with gDNA Eraser**	TaKaRa	RR047B
**RNeasy Mini Kit**	Qiagen	74104
**QIAquick PCR Purification Kit**	Qiagen	28104
